# Transcriptome-wide association study of breast cancer risk by estrogen-receptor status

**DOI:** 10.1002/gepi.22288

**Published:** 2020-03-01

**Authors:** Helian Feng, Alexander Gusev, Bogdan Pasaniuc, Lang Wu, Jirong Long, Zomoroda Abu-full, Kristiina Aittomäki, Irene L. Andrulis, Hoda Anton-Culver, Antonis C. Antoniou, Adalgeir Arason, Volker Arndt, Kristan J. Aronson, Banu K. Arun, Ella Asseryanis, Paul L. Auer, Jacopo Azzollini, Judith Balmaña, Rosa B. Barkardottir, Daniel R. Barnes, Daniel Barrowdale, Matthias W. Beckmann, Sabine Behrens, Javier Benitez, Marina Bermisheva, Katarzyna Białkowska, Ana Blanco, Carl Blomqvist, Bram Boeckx, Natalia V. Bogdanova, Stig E. Bojesen, Manjeet K. Bolla, Bernardo Bonanni, Ake Borg, Hiltrud Brauch, Hermann Brenner, Ignacio Briceno, Annegien Broeks, Thomas Brüning, Barbara Burwinkel, Qiuyin Cai, Trinidad Caldés, Maria A. Caligo, Ian Campbell, Sander Canisius, Daniele Campa, Brian D. Carter, Jonathan Carter, Jose E. Castelao, Jenny Chang-Claude, Stephen J. Chanock, Hans Christiansen, Wendy K. Chung, Kathleen B. M. Claes, Christine L. Clarke, GEMO Study Collaborators, EMBRACE Collaborators, GC-HBOC study Collaborators, Fergus J. Couch, Angela Cox, Simon S. Cross, Cezary Cybulski, Kamila Czene, Mary B. Daly, Miguel de la Hoya, Kim De Leeneer, Joe Dennis, Peter Devilee, Orland Diez, Susan M. Domchek, Thilo Dörk, Isabel dos-Santos-Silva, Alison M. Dunning, Miriam Dwek, Diana M. Eccles, Bent Ejlertsen, Carolina Ellberg, Christoph Engel, Mikael Eriksson, Peter A. Fasching, Olivia Fletcher, Henrik Flyger, Florentia Fostira, Eitan Friedman, Lin Fritschi, Debra Frost, Marike Gabrielson, Patricia A. Ganz, Susan M. Gapstur, Judy Garber, Montserrat García-Closas, José A. García-Sáenz, Mia M. Gaudet, Graham G. Giles, Gord Glendon, Andrew K. Godwin, Mark S. Goldberg, David E. Goldgar, Anna González-Neira, Mark H. Greene, Jacek Gronwald, Pascal Guénel, Christopher A. Haiman, Per Hall, Ute Hamann, Christopher Hake, Wei He, Jane Heyworth, Frans B.L. Hogervorst, Antoinette Hollestelle, Maartje J. Hooning, Robert N. Hoover, John L. Hopper, Guanmengqian Huang, Peter J. Hulick, Keith Humphreys, Evgeny N. Imyanitov, ABCTB Investigators, HEBON Investigators, BCFR Investigators, OCGN Investigators, Claudine Isaacs, Milena Jakimovska, Anna Jakubowska, Paul James, Ramunas Janavicius, Rachel C. Jankowitz, Esther M. John, Nichola Johnson, Vijai Joseph, Audrey Jung, Beth Y. Karlan, Elza Khusnutdinova, Johanna I. Kiiski, Irene Konstantopoulou, Vessela N. Kristensen, Yael Laitman, Diether Lambrechts, Conxi Lazaro, Dominique Leroux, Goska Leslie, Jenny Lester, Fabienne Lesueur, Noralane Lindor, Sara Lindström, Wing-Yee Lo, Jennifer T. Loud, Jan Lubiński, Enes Makalic, Arto Mannermaa, Mehdi Manoochehri, Siranoush Manoukian, Sara Margolin, John W.M. Martens, Maria E. Martinez, Laura Matricardi, Tabea Maurer, Dimitrios Mavroudis, Lesley McGuffog, Alfons Meindl, Usha Menon, Kyriaki Michailidou, Pooja M. Kapoor, Austin Miller, Marco Montagna, Fernando Moreno, Lidia Moserle, Anna M. Mulligan, Taru A. Muranen, Katherine L. Nathanson, Susan L. Neuhausen, Heli Nevanlinna, Ines Nevelsteen, Finn C. Nielsen, Liene Nikitina-Zake, Kenneth Offit, Edith Olah, Olufunmilayo I. Olopade, Håkan Olsson, Ana Osorio, Janos Papp, Tjoung-Won Park-Simon, Michael T. Parsons, Inge S. Pedersen, Ana Peixoto, Paolo Peterlongo, Julian Peto, Paul D.P. Pharoah, Kelly-Anne Phillips, Dijana Plaseska-Karanfilska, Bruce Poppe, Nisha Pradhan, Karolina Prajzendanc, Nadege Presneau, Kevin Punie, Katri Pylkäs, Paolo Radice, Johanna Rantala, Muhammad Usman Rashid, Gad Rennert, Harvey A Risch, Mark Robson, Atocha Romero, Emmanouil Saloustros, Dale P. Sandler, Catarina Santos, Elinor J. Sawyer, Marjanka K. Schmidt, Daniel F. Schmidt, Rita K. Schmutzler, Minouk J. Schoemaker, Rodney J Scott, Priyanka Sharma, Xiao-Ou Shu, Jacques Simard, Christian F. Singer, Anne-Bine Skytte, Penny Soucy, Melissa C. Southey, John J. Spinelli, Amanda B. Spurdle, Jennifer Stone, Anthony J. Swerdlow, William J. Tapper, Jack A. Taylor, Manuel R. Teixeira, Mary Beth Terry, Alex Teulé, Mads Thomassen, Kathrin Thöne, Darcy L. Thull, Marc Tischkowitz, Amanda E. Toland, Rob A. E. M. Tollenaar, Diana Torres, Thérèse Truong, Nadine Tung, Celine M. Vachon, Christi J. van Asperen, Ans M. W. van den Ouweland, Elizabeth J. van Rensburg, Ana Vega, Alessandra Viel, Paula Vieiro-Balo, Qin Wang, Barbara Wappenschmidt, Clarice R. Weinberg, Jeffrey N. Weitzel, Camilla Wendt, Robert Winqvist, Xiaohong R. Yang, Drakoulis Yannoukakos, Argyrios Ziogas, Roger L. Milne, Douglas F. Easton, Georgia Chenevix-Trench, Wei Zheng, Peter Kraft, Xia Jiang

**Affiliations:** 1 Program in Genetic Epidemiology and Statistical Genetics, Harvard T. H. Chan School of Public Health, Boston, Massachusetts; 2 Department of Epidemiology, Harvard T. H. Chan School of Public Health, Boston, Massachusetts; 3 Department of Biostatistics, Harvard T. H. Chan School of Public Health, Boston, Massachusetts; 4 Dana-Farber Cancer Institute, Boston, Massachusetts; 5 UCLA Path & Lab Med, Los Angeles, California; 6 Epidemiology Program, University of Hawaii Cancer Center, Honolulu, Hawaii; 7 Division of Epidemiology, Department of Medicine, Vanderbilt Epidemiology Center, Vanderbilt-Ingram Cancer Center, Vanderbilt University School of Medicine, Nashville, Tennessee; 8 Clalit National Cancer Control Center, Carmel Medical Center and Technion Faculty of Medicine, Haifa, Israel; 9 Department of Clinical Genetics, Helsinki University Hospital, University of Helsinki, Helsinki, Finland; 10 Fred A, Litwin Center for Cancer Genetics, Lunenfeld-Tanenbaum Research Institute of Mount Sinai Hospital, Toronto, Ontario, Canada; 11 Department of Molecular Genetics, University of Toronto, Toronto, Ontario, Canada; 12 Department of Epidemiology, Genetic Epidemiology Research Institute, University of California Irvine, Irvine, California; 13 Centre for Cancer Genetic Epidemiology, Department of Public Health and Primary Care, University of Cambridge, Cambridge, UK; 14 Department of Pathology, Landspitali University Hospital, Reykjavik, Iceland; 15 BMC (Biomedical Centre), Faculty of Medicine, University of Iceland, Reykjavik, Iceland; 16 Division of Clinical Epidemiology and Aging Research, German Cancer Research Center (DKFZ), Heidelberg, Germany; 17 Department of Public Health Sciences, and Cancer Research Institute, Queen’s University, Kingston, Ontario, Canada; 18 Department of Breast Medical Oncology, University of Texas MD Anderson Cancer Center, Houston, Texas; 19 Department of OB/GYN and Comprehensive Cancer Center, Medical University of Vienna, Vienna, Austria; 20 Cancer Prevention Program, Fred Hutchinson Cancer Research Center, Seattle, Washington; 21 Zilber School of Public Health, University of Wisconsin-Milwaukee, Milwaukee, Wisconsin; 22 Unit of Medical Genetics, Department of Medical Oncology and Hematology, Fondazione IRCCS Istituto Nazionale dei Tumori di Milano, Milan, Italy; 23 High Risk and Cancer Prevention Group, Vall d’Hebron Institute of Oncology, Barcelona, Spain; 24 Department of Gynecology and Obstetrics, Comprehensive Cancer Center ER-EMN, University Hospital Erlangen, Friedrich-Alexander-University Erlangen-Nuremberg, Erlangen, Germany; 25 Division of Cancer Epidemiology, German Cancer Research Center (DKFZ), Heidelberg, Germany; 26 Centro de Investigaci—n en Red de Enfermedades Raras (CIBERER), Madrid, Spain; 27 Human Cancer Genetics Programme, Spanish National Cancer Research Centre (CNIO), Madrid, Spain; 28 Institute of Biochemistry and Genetics, Ufa Federal Research Centre of the Russian Academy of Sciences, Ufa, Russia; 29 Department of Genetics and Pathology, Pomeranian Medical University, Szczecin, Poland; 30 Fundaci—n Pœblica Galega Medicina Xen—mica, Santiago De Compostela, Spain; 31 Instituto de Investigacion Sanitaria de Santiago de Compostela, Santiago de Compostela, Spain; 32 Department of Oncology, Helsinki University Hospital, University of Helsinki, Helsinki, Finland; 33 Department of Oncology, University Hospital, Karolinska Institute, Stockholm, Sweden; 34 VIB Center for Cancer Biology, VIB, Leuven, Belgium; 35 Laboratory for Translational Genetics, Department of Human Genetics, University of Leuven, Leuven, Belgium; 36 Department of Radiation Oncology, Hannover Medical School, Hannover, Germany; 37 Gynaecology Research Unit, Hannover Medical School, Hannover, Germany; 38 NN Alexandrov Research Institute of Oncology and Medical Radiology, Minsk, Belarus; 39 Copenhagen General Population Study, Herlev and Gentofte Hospital, Copenhagen University Hospital, Herlev, Denmark; 40 Department of Clinical Biochemistry, Herlev and Gentofte Hospital, Copenhagen University Hospital, Herlev, Denmark; 41 Faculty of Health and Medical Sciences, University of Copenhagen, Copenhagen, Denmark; 42 Division of Cancer Prevention and Genetics, IEO, European Institute of Oncology IRCCS, Milan, Italy; 43 Department of Oncology, Lund University and Skåne University Hospital, Lund, Sweden; 44 Dr. Margarete Fischer-Bosch-Institute of Clinical Pharmacology, Stuttgart, Germany; 45 iFIT-Cluster of Excellence, University of Tuebingen, Tuebingen, Germany; 46 German Cancer Consortium (DKTK), German Cancer Research Center (DKFZ), Heidelberg, Germany; 47 Division of Preventive Oncology, German Cancer Research Center (DKFZ) and National Center for Tumor Diseases (NCT), Heidelberg, Germany; 48 Institute of Human Genetics, Pontificia Universidad Javeriana, Bogota, Colombia; 49 Medical Faculty, Universidad de La Sabana, Bogota, Colombia; 50 Division of Molecular Pathology, The Netherlands Cancer Institute—Antoni van Leeuwenhoek Hospital, Amsterdam, The Netherlands; 51 Institute for Prevention and Occupational Medicine of the German Social Accident Insurance, Institute of the Ruhr University Bochum (IPA), Bochum, Germany; 52 Molecular Epidemiology Group, German Cancer Research Center (DKFZ), Heidelberg, Germany; 53 Molecular Biology of Breast Cancer, University Womens Clinic Heidelberg, University of Heidelberg, Heidelberg, Germany; 54 Medical Oncology Department, Hospital Cl’nico San Carlos, Instituto de Investigaci—n Sanitaria San Carlos (IdISSC), Centro Investigación Biomédica en Red de Cáncer (CIBERONC), Madrid, Spain; 55 Section of Molecular Genetics, Dept, of Laboratory Medicine, University Hospital of Pisa, Pisa, Italy; 56 Research Department, Peter MacCallum Cancer Center, Melbourne, Victoria, Australia; 57 Sir Peter MacCallum Department of Oncology, The University of Melbourne, Melbourne, Victoria, Australia; 58 Division of Molecular Carcinogenesis, The Netherlands Cancer Institute—Antoni van Leeuwenhoek hospital, Amsterdam, The Netherlands; 59 Genomic Epidemiology Group, German Cancer Research Center (DKFZ), Heidelberg, Germany; 60 Behavioral and Epidemiology Research Group, American Cancer Society, Atlanta, Georgia; 61 Department of Gynaecological Oncology, Chris OÕBrien Lifehouse and The University of Sydney, Camperdown, New South Wales, Australia; 62 Oncology and Genetics Unit, Instituto de Investigacion Sanitaria Galicia Sur (IISGS), Xerencia de Xestion Integrada de Vigo-SERGAS, Vigo, Spain; 63 Cancer Epidemiology Group, University Cancer Center Hamburg (UCCH), University Medical Center Hamburg-Eppendorf, Hamburg, Germany; 64 Division of Cancer Epidemiology and Genetics, Department of Health and Human Services, National Cancer Institute, National Institutes of Health, Bethesda, Maryland; 65 Departments of Pediatrics and Medicine, Columbia University, New York, New York; 66 Centre for Medical Genetics, Ghent University, Gent, Belgium; 67 Westmead Institute for Medical Research, University of Sydney, Sydney, New South Wales, Australia; 68 Department of Tumour Biology, INSERM U830, Paris, France; 69 Institut Curie, Paris, France; 70 Mines ParisTech, Fontainebleau, France; 71 Center for Hereditary Breast and Ovarian Cancer, Faculty of Medicine and University Hospital Cologne, University of Cologne, Cologne, Germany; 72 Department of Laboratory Medicine and Pathology, Mayo Clinic, Rochester, Minnesota; 73 Department of Oncology and Metabolism, Sheffield Institute for Nucleic Acids (SInFoNiA), University of Sheffield, Sheffield, UK; 74 Academic Unit of Pathology, Department of Neuroscience, University of Sheffield, Sheffield, UK; 75 Department of Medical Epidemiology and Biostatistics, Karolinska Institutet, Stockholm, Sweden; 76 Department of Clinical Genetics, Fox Chase Cancer Center, Philadelphia, Pennsylvania; 77 Department of Pathology, Leiden University Medical Center, Leiden, The Netherlands; 78 Department of Human Genetics, Leiden University Medical Center, Leiden, The Netherlands; 79 Hereditary Cancer Genetics Group, Area of Clinical and Molecular Genetics, Vall dHebron Institute of Oncology (VHIO), University Hospital Vall d’Hebron, Barcelona, Spain; 80 Clinical and Molecular Genetics Area, University Hospital Vall dHebron, Barcelona, Spain; 81 Department of Medicine, Abramson Cancer Center, Perelman School of Medicine at the University of Pennsylvania, Philadelphia, Pennsylvania; 82 Department of Non-Communicable Disease Epidemiology, London School of Hygiene and Tropical Medicine, London, UK; 83 Centre for Cancer Genetic Epidemiology, Department of Oncology, University of Cambridge, Cambridge, UK; 84 Department of Biomedical Sciences, Faculty of Science and Technology, University of Westminster, London, UK; 85 Cancer Sciences Academic Unit, Faculty of Medicine, University of Southampton, Southampton, UK; 86 Department of Oncology, Rigshospitalet, Copenhagen University Hospital, Copenhagen, Denmark; 87 Department of Cancer Epidemiology, Clinical Sciences, Lund University, Lund, Sweden; 88 Institute for Medical Informatics, Statistics and Epidemiology, University of Leipzig, Leipzig, Germany; 89 LIFE - Leipzig Research Centre for Civilization Diseases, University of Leipzig, Leipzig, Germany; 90 David Geffen School of Medicine, Department of Medicine Division of Hematology and Oncology, University of California at Los Angeles, Los Angeles, California; 91 The Breast Cancer Now Toby Robins Research Centre, The Institute of Cancer Research, London, UK; 92 Department of Breast Surgery, Herlev and Gentofte Hospital, Copenhagen University Hospital, Herlev, Denmark; 93 Molecular Diagnostics Laboratory, INRASTES, National Centre for Scientific Research ‘Demokritos’, Athens, Greece; 94 The Susanne Levy Gertner Oncogenetics Unit, Chaim Sheba Medical Center, Ramat Gan, Israel; 95 Sackler Faculty of Medicine, Tel Aviv University, Ramat Aviv, Israel; 96 School of Public Health, Curtin University, Perth, Western Australia, Australia; 97 Schools of Medicine and Public Health, Division of Cancer Prevention & Control Research, Jonsson Comprehensive Cancer Centre, UCLA, Los Angeles, California; 98 Cancer Risk and Prevention Clinic, Dana-Farber Cancer Institute, Boston, Massachusetts; 99 Division of Genetics and Epidemiology, Institute of Cancer Research, London, UK; 100 Cancer Epidemiology Division, Cancer Council Victoria, Melbourne, Victoria, Australia; 101 Centre for Epidemiology and Biostatistics, Melbourne School of Population and Global Health, The University of Melbourne, Melbourne, Victoria, Australia; 102 Department of Epidemiology and Preventive Medicine, Monash University, Melbourne, Victoria, Australia; 103 Department of Pathology and Laboratory Medicine, Kansas University Medical Center, Kansas City, Kanas; 104 Department of Medicine, McGill University, Montreal, Quebec, Canada; 105 Division of Clinical Epidemiology, Royal Victoria Hospital, McGill University, Montreal, Quebec, Canada; 106 Department of Dermatology, Huntsman Cancer Institute, University of Utah School of Medicine, Salt Lake City, Utah; 107 Clinical Genetics Branch, Division of Cancer Epidemiology and Genetics, National Cancer Institute, Bethesda, Maryland; 108 Cancer & Environment Group, Center for Research in Epidemiology and Population Health (CESP), INSERM, University Paris-Sud, University Paris-Saclay, Villejuif, France; 109 Department of Preventive Medicine, Keck School of Medicine, University of Southern California, Los Angeles, California; 110 Department of Oncology, Sšdersjukhuset, Stockholm, Sweden; 111 Molecular Genetics of Breast Cancer, German Cancer Research Center (DKFZ), Heidelberg, Germany; 112 City of Hope Clinical Cancer Genetics Community Research Network, Duarte, California; 113 School of Population and Global Health, The University of Western Australia, Perth, Western Australia, Australia; 114 Family Cancer Clinic, The Netherlands Cancer Institute—Antoni van Leeuwenhoek Hospital, Amsterdam, The Netherlands; 115 Department of Medical Oncology, Family Cancer Clinic, Erasmus MC Cancer Institute, Rotterdam, The Netherlands; 116 Center for Medical Genetics, NorthShore University HealthSystem, Evanston, Illinois; 117 The University of Chicago Pritzker School of Medicine, Chicago, Illinois; 118 NN Petrov Institute of Oncology, St. Petersburg, Russia; 119 Australian Breast Cancer Tissue Bank, Westmead Institute for Medical Research, University of Sydney, Sydney, New South Wales, Australia; 120 The Hereditary Breast and Ovarian Cancer Research Group Netherlands (HEBON), Coordinating Center, The Netherlands Cancer Institute, Amsterdam, The Netherlands; 121 Department of Medicine, Division of Oncology, Stanford Cancer Institute, Stanford University School of Medicine, Stanford, California; 122 Ontario Cancer Genetics Network, Lunenfeld-Tanenbaum Research Institute of Mount Sinai Hospital, Toronto, Ontario, Canada; 123 Lombardi Comprehensive Cancer Center, Georgetown University, Washington, District of Columbia; 124 Research Centre for Genetic Engineering and Biotechnology ‘Georgi D, Efremov’, Macedonian Academy of Sciences and Arts, Skopje Republic of North Macedonia, North Macedonia; 125 Independent Laboratory of Molecular Biology and Genetic Diagnostics, Pomeranian Medical University, Szczecin, Poland; 126 Parkville Familial Cancer Centre, Peter MacCallum Cancer Center, Melbourne, Victoria, Australia; 127 State Research Institute Innovative Medicine Center, Vilnius, Lithuania; 128 Department of Medicine, Division of Hematology/Oncology, UPMC Hillman Cancer Center, University of Pittsburgh School of Medicine, Pittsburgh, Pennsylvania; 129 Clinical Genetics Research Lab, Department of Cancer Biology and Genetics, Memorial Sloan-Kettering Cancer Center, New York, New York; 130 David Geffen School of Medicine, Department of Obstetrics and Gynecology, University of California, Los Angeles, California; 131 Department of Genetics and Fundamental Medicine, Bashkir State Medical University, Ufa, Russia; 132 Department of Obstetrics and Gynecology, Helsinki University Hospital, University of Helsinki, Helsinki, Finland; 133 Department of Cancer Genetics, Institute for Cancer Research, Oslo University Hospital-Radiumhospitalet, Oslo, Norway; 134 Institute of Clinical Medicine, Faculty of Medicine, University of Oslo, Oslo, Norway; 135 Molecular Diagnostic Unit, Hereditary Cancer Program, ICO-IDIBELL (Bellvitge Biomedical Research Institute, Catalan Institute of Oncology), CIBERONC, Barcelona, Spain; 136 Departement de Ge netique, CHU de Grenoble, Grenoble, France; 137 Genetic Epidemiology of Cancer Team, Inserm U900, Paris, France; 138 Department of Health Sciences Research, Mayo Clinic, Scottsdale, Arizona; 139 Department of Epidemiology, University of Washington School of Public Health, Seattle, Washington; 140 Public Health Sciences Division, Fred Hutchinson Cancer Research Center, Seattle, Washington; 141 Translational Cancer Research Area, University of Eastern Finland, Kuopio, Finland; 142 Institute of Clinical Medicine, Pathology and Forensic Medicine, University of Eastern Finland, Kuopio, Finland; 143 Imaging Center, Department of Clinical Pathology, Kuopio University Hospital, Kuopio, Finland; 144 Department of Clinical Science and Education, Sšdersjukhuset, Karolinska Institutet, Stockholm, Sweden; 145 Moores Cancer Center, University of California San Diego, La Jolla, California; 146 Department of Family Medicine and Public Health, University of California San Diego, La Jolla, California; 147 Immunology and Molecular Oncology Unit, Veneto Institute of Oncology ÊIOV—IRCCS, Padua, Italy; 148 Department of Medical Oncology, University Hospital of Heraklion, Heraklion, Greece; 149 Department of Gynecology and Obstetrics, Ludwig Maximilian University of Munich, Munich, Germany; 150 MRC Clinical Trials Unit at UCL, Institute of Clinical Trials & Methodology, University College London, London, UK; 151 Department of Electron Microscopy/Molecular Pathology and The Cyprus School of Molecular Medicine, The Cyprus Institute of Neurology & Genetics, Nicosia, Cyprus; 152 Faculty of Medicine, University of Heidelberg, Heidelberg, Germany; 153 NRG Oncology, Statistics and Data Management Center, Roswell Park Cancer Institute, Buffalo, New York; 154 Department of Laboratory Medicine and Pathobiology, University of Toronto, Toronto, Ontario, Canada; 155 Laboratory Medicine Program, University Health Network, Toronto, Ontario, Canada; 156 Department of Population Sciences, Beckman Research Institute of City of Hope, Duarte, California; 157 Leuven Multidisciplinary Breast Center, Department of Oncology, Leuven Cancer Institute, University Hospitals Leuven, Leuven, Belgium; 158 Center for Genomic Medicine, Rigshospitalet, Copenhagen University Hospital, Copenhagen, Denmark; 159 Latvian Biomedical Research and Study Centre, Riga, Latvia; 160 Clinical Genetics Service, Department of Medicine, Memorial Sloan-Kettering Cancer Center, New York, New York; 161 Department of Molecular Genetics, National Institute of Oncology, Budapest, Hungary; 162 Center for Clinical Cancer Genetics, The University of Chicago, Chicago, Illinois; 163 Department of Genetics and Computational Biology, QIMR Berghofer Medical Research Institute, Brisbane, Queensland, Australia; 164 Section of Molecular Diagnostics, Clinical Biochemistry, Aalborg University Hospital, Aalborg, Denmark; 165 Department of Genetics, Portuguese Oncology Institute, Porto, Portugal; 166 Genome Diagnostics Program, IFOM—The FIRC (Italian Foundation for Cancer Research) Institute of Molecular Oncology, Milan, Italy; 167 Department of Medicine Oncology, Peter MacCallum Cancer Centre, Melbourne, Victoria, Australia; 168 Laboratory of Cancer Genetics and Tumor Biology, Cancer and Translational Medicine Research Unit, Biocenter Oulu, University of Oulu, Oulu, Finland; 169 Laboratory of Cancer Genetics and Tumor Biology, Northern Finland Laboratory Centre Oulu, Oulu, Finland; 170 Unit of Molecular Bases of Genetic Risk and Genetic Testing, Department of Research, Fondazione IRCCS Istituto Nazionale dei Tumori (INT), Milan, Italy; 171 Clinical Genetics, Karolinska Institutet, Stockholm, Sweden; 172 Department of Basic Sciences, Shaukat Khanum Memorial Cancer Hospital and Research Centre (SKMCH & RC), Lahore, Pakistan; 173 Department of Chronic Disease Epidemiology, Yale School of Public Health, New Haven, Connecticut; 174 Medical Oncology Department, Hospital Universitario Puerta de Hierro, Madrid, Spain; 175 Department of Oncology, University Hospital of Larissa, Larissa, Greece; 176 Epidemiology Branch, National Institute of Environmental Health Sciences, NIH, Research Triangle Park, North Carolina; 177 Research Oncology, GuyÕs Hospital, King’s College London, London, UK; 178 Division of Psychosocial Research and Epidemiology, The Netherlands Cancer Institute—Antoni van Leeuwenhoek Hospital, Amsterdam, The Netherlands; 179 Faculty of Information Technology, Monash University, Melbourne, Victoria, Australia; 180 Center for Molecular Medicine Cologne (CMMC), Faculty of Medicine and University Hospital Cologne, University of Cologne, Cologne, Germany; 181 Division of Molecular Medicine, Pathology North, John Hunter Hospital, Newcastle, New South Wales, Australia; 182 Discipline of Medical Genetics, School of Biomedical Sciences and Pharmacy, Faculty of Health, University of Newcastle, Callaghan, New South Wales, Australia; 183 Hunter Medical Research Institute, John Hunter Hospital, Newcastle, New South Wales, Australia; 184 Department of Internal Medicine, Division of Medical Oncology, University of Kansas Medical Center, Westwood, Kanas; 185 Genomics Center, Centre Hospitalier Universitaire de Quebec–Universite Laval, Research Center, Quebec City, Qubec, Canada; 186 Department of Clinical Genetics, Aarhus University Hospital, Aarhus N, Denmark; 187 Precision Medicine, School of Clinical Sciences at Monash Health, Monash University, Clayton, Victoria, Australia; 188 Department of Clinical Pathology, The University of Melbourne, Melbourne, Victoria, Australia; 189 Population Oncology, BC Cancer, Vancouver, British of Columbia, Canada; 190 School of Population and Public Health, University of British Columbia, Vancouver, British of Columbia, Canada; 191 The Curtin UWA Centre for Genetic Origins of Health and Disease, Curtin University and University of Western Australia, Perth, Western Australia, Australia; 192 Division of Breast Cancer Research, The Institute of Cancer Research, London, UK; 193 Faculty of Medicine, University of Southampton, Southampton, UK; 194 Epigenetic and Stem Cell Biology Laboratory, National Institute of Environmental Health Sciences, NIH, Research Triangle Park, North Carolina; 195 Biomedical Sciences Institute (ICBAS), University of Porto, Porto, Portugal; 196 Department of Epidemiology, Mailman School of Public Health, Columbia University, New York, New York; 197 Genetic Counseling Unit, Hereditary Cancer Program, IDIBELL (Bellvitge Biomedical Research Institute), Catalan Institute of Oncology, CIBERONC, Barcelona, Spain; 198 Department of Clinical Genetics, Odense University Hospital, Odence C, Denmark; 199 Department of Medicine, Magee-Womens Hospital, University of Pittsburgh School of Medicine, Pittsburgh, Pennsylvania; 200 Program in Cancer Genetics, Departments of Human Genetics and Oncology, McGill University, Montreal, Quebec, Canada; 201 Department of Medical Genetics, University of Cambridge, Cambridge, UK; 202 Department of Cancer Biology and Genetics, The Ohio State University, Columbus, Ohio; 203 Department of Surgery, Leiden University Medical Center, Leiden, The Netherlands; 204 Department of Medical Oncology, Beth Israel Deaconess Medical Center, Boston, Massachusetts; 205 Department of Health Science Research, Division of Epidemiology, Mayo Clinic, Rochester, Minnesota; 206 Department of Clinical Genetics, Leiden University Medical Center, Leiden, The Netherlands; 207 Department of Clinical Genetics, Erasmus University Medical Center, Rotterdam, The Netherlands; 208 Department of Genetics, University of Pretoria, Arcadia, South Africa; 209 Division of Functional Onco-genomics and Genetics, Centro di Riferimento Oncologico di Aviano (CRO), IRCCS, Aviano, Italy; 210 Hospital Clínico Universitario (SERGAS), Universidad de Santiago de Compostela, CIMUS, Santiago de Compostela, España; 211 Biostatistics and Computational Biology Branch, National Institute of Environmental Health Sciences, NIH, Research Triangle Park, North Carolina; 212 Clinical Cancer Genomics, City of Hope, Duarte, California

**Keywords:** breast cancer subtype, causal gene, GWAS, TWAS

## Abstract

Previous transcriptome-wide association studies (TWAS) have identified breast cancer risk genes by integrating data from expression quantitative loci and genome-wide association studies (GWAS), but analyses of breast cancer subtype-specific associations have been limited. In this study, we conducted a TWAS using gene expression data from GTEx and summary statistics from the hitherto largest GWAS meta-analysis conducted for breast cancer overall, and by estrogen receptor subtypes (ER+ and ER−). We further compared associations with ER+ and ER− subtypes, using a case-only TWAS approach. We also conducted multigene conditional analyses in regions with multiple TWAS associations. Two genes, *STXBP4* and *HIST2H2BA*, were specifically associated with ER+ but not with ER− breast cancer. We further identified 30 TWAS-significant genes associated with overall breast cancer risk, including four that were not identified in previous studies. Conditional analyses identified single independent breast-cancer gene in three of six regions harboring multiple TWAS-significant genes. Our study provides new information on breast cancer genetics and biology, particularly about genomic differences between ER+ and ER− breast cancer.

## 1 | INTRODUCTION

Breast cancer is the most common malignancy among women worldwide (Bray et al., 2018). The disease has a strong inherited component (Beggs & Hodgson, 2009); linkage studies have identified infrequent mutations in *BRCA1/2* (Easton et al., 2007; Seal et al., 2006; Turnbull et al., 2010) and genome-wide association studies (GWAS) have identified 177 susceptibility loci to date (Michailidou et al., 2017). However, these GWAS-discovered variants explain only 18% of the familial relative risk of breast cancer. Moreover, the causal mechanism driving GWAS associations remains largely unknown, as many variants are located in noncoding or intergenic regions, and are not in strong linkage disequilibrium (LD) with known protein-coding variants (Beggs & Hodgson, 2009; Michailidou et al., 2015).

Breast cancer is a heterogeneous disease consisting of several well-established subtypes. One of the most important markers of breast cancer subtypes is estrogen receptor (ER) status. ER+ and ER− tumors differ in etiology (X. R. Yang, Chang-Claude, et al., 2011), genetic predisposition (Mavaddat, Antoniou, Easton, & Garcia-Closas, 2010), and clinical behavior (Blows et al., 2010). ER− tumor occurs more often among younger women, and patients are more likely to carry *BRCA1* pathogenic variants (Atchley et al., 2008; Garcia-Closas et al., 2013). ER− tumor also has worse short-term prognosis. Among the 177 GWAS-identified breast cancer-associated single nucleotide polymorphisms (SNPs), around 50 are more strongly associated with ER+ disease and 20 are more strongly associated with ER− disease (Michailidou et al., 2017; Milne et al., 2017).

SNPs associated with complex traits are more likely to be in regulatory regions than in protein-coding regions, and many of these SNPs are also associated with expression levels of nearby genes (Nicolae et al., 2010). For example, breast cancer GWAS-identified variants at 6q25.1 regulate *ESR1*, but also coregulate other local genes such as *RMND1*, *ARMT1*, and *CCDC170* (Dunning et al., 2016, p. 1). These results suggest that by integrating genotype, phenotype, and gene expression, we can identify novel trait-associated genes and understand biological mechanisms. However, due to costs and tissue availability, acquiring GWAS and gene expression data for the same set of individuals remains challenging.

A recently published approach, referred to as transcriptome-wide association study (TWAS; Gamazon et al., 2015; Gusev et al., 2016), overcomes these difficulties by using a relatively small set of reference individuals for whom both gene expression and SNPs have been measured to impute the *cis*-genetic component of expression for a much larger set of individuals from their GWAS summary statistics. The association between the predicted gene expression and traits can then be tested. This method has been shown to have greater power relative to GWAS; and has identified 1,196 trait-associated genes across 30 complex traits in a recently performed multitissue TWAS (Mancuso et al., 2017).

To date, three TWAS of breast cancer have been conducted (Gao, Pierce, Olopade, Im, & Huo, 2017; Hoffman et al., 2017; Wu et al., 2018). A fourth study linked expression quantitative loci (eQTL) data across multiple tissues and breast cancer GWAS results using EUGENE, a statistical approach that sums evidence for association with disease across eQTLs regardless of directionality. That study then tested EUGENE-significant genes using a TWAS statistic, which does take directionality into account (Ferreira et al., 2019). The two earliest TWAS used GWAS data from the National Cancer Institute’s “Up for a Challenge” competition, which included data from 12,100 breast cancer cases (of which 3,900 had ER− disease) and 11,400 controls, as well as eQTL data from breast tissue and whole blood from the GTEx and DGN projects (Gao et al., 2017; Hoffman et al., 2017). The subsequent TWAS by Wu et al. (2018) and the EUGENE analysis by Ferreira et al. (2019) used results from a much larger GWAS conducted by the Breast Cancer Association Consortium (BCAC), which included 122,977 cases (of which 21,468 had ER− disease) and 105,974 controls. Together, these four studies have identified 59 genes whose predicted expression levels are associated with risk of overall breast cancer, and five associated with risk of ER− disease. Of these 64 genes, 30 are at loci not previously identified by breast cancer GWAS.

These previous TWAS largely focused on overall breast cancer risk. Analyses of ER− disease either were conducted using a small sample size (Gao et al., 2017) or did not scan all genes using a directional TWAS approach (Ferreira et al., 2019). Moreover, none of the previous analyses considered ER+ disease specifically or examined differences in association between predicted gene expression and ER+ versus ER− disease.

The interpretation of TWAS results is not straight-forward (Wainberg et al., 2019). Specifically, TWAS statistic by itself cannot distinguish between a mediated effect (SNPs influence breast cancer risk by changing the expression of the tested gene), pleiotropy (SNPs associated with gene expression also influence breast cancer risk through another mechanism), or colocalization (SNPs associated with gene expression are in LD with other SNPs that influence breast cancer risk through another mechanism). Previous studies have conducted limited sensitivity analyses (e.g., Wu et al., 2018 and Ferreira et al., 2019 conditioned the TWAS tests on lead GWAS SNPs), but the genetic architecture at TWAS-identified loci remains largely unclear.

In the current analysis, we complement previous work by conducting a TWAS for overall breast cancer and for ER+ and ER− subtypes. We also applied a case-only TWAS test to identify predicted transcript levels that were differentially associated with ER+ and ER− disease. We conducted expanded sensitivity analyses, conditioning on multiple TWAS-significant genes in a region to account for possible confounding due to LD (colocalization). We chose to focus on the expression of normal breast tissue of European ancestry women to maximize specificity and identify good targets for near-term follow-up experiments in mammary cells. One advantage of using a biologically relevant tissue is that it both increases the a priori plausibility of observed associations and increases the likelihood that genes with observed associations will be expressed and influence tumor development in cells from the target tissue. We have reproduced previous results (Ferreira et al., 2019; Wu et al., 2018) and provided evidence regarding the independent associations of multiple genes in regions containing one or more TWAS-significant genes. We also identified genes with subtype-specific associations, highlighting different biological mechanisms likely underlying the disease subtypes.

## 2 | MATERIAL AND METHODS

### 2.1 | Gene expression reference panel

The transcriptome and high-density genotyping data used to build the gene expression model (reference panel) were retrieved from GTEx (GTEx Consortium, 2015), a consortium collected high-quality gene expression RNA-seq data across 44 body sites from 449 donors, and genome-wide genetic information. For the current study, we included 67 women of European ancestry who provided normal breast mammary tissues. RNA samples extracted from tissues were sequenced to generate data on 12,696 transcripts. Genomic DNA samples were genotyped using Illumina OMNI 5 M or 2.5 M arrays, processed with a standard GTEx protocol. Briefly, SNPs with call rates <98%, with differential missingness between the two array experiments (5 M or 2.5 M), with Hardy-Weinberg equilibrium *p* < 10^−6^ or showing batch effects were excluded. The genotypes were then imputed to the Haplotype Reference Consortium reference panel (McCarthy et al., 2016) using Minimac3 for imputation and SHAPEIT for pre-phasing (Delaneau, Marchini, & Zagury, 2011; Howie, Donnelly, & Marchini, 2009). Only SNPs with high imputation quality (*r*^2^≥ .8), minor allele frequency (MAF) ≥0.05, and were included in the Hap-Map Phase 2 version were used to build the expression prediction models.

### 2.2 | Breast cancer meta-GWAS data

The GWAS breast cancer summary-level data were mainly provided by the Breast Cancer Association Consortium (BCAC; Michailidou et al., 2017), as well as the Consortium of Investigators of Modifiers of *BRCA1/2* (CIMBA). BCAC conducted the largest breast cancer meta-GWAS to date (referred as the overall breast cancer GWAS analysis). The BCAC included 122,977 cases and 105,974 controls of European ancestry. Among these, 46,785 cases and 42,892 controls were genotyped using the Illumina iSelect genotyping array (iCOGS) on 211,155 SNPs; and 61,282 cases and 45,494 controls were genotyped using the Illumina OncoArray on 570,000 SNPs (Yovel, Franz, Stilz, & Schnitzler, 2008). The study also included data from 11 other GWAS on 14,910 cases and 17,588 controls. Genetic data for all individual participating studies were imputed to the 1000 Genomes Project Phase 3 v5 EUR reference panel Logistic regression was fitted to estimate per-allele odds ratios (ORs), adjusting for country and top principal components (PCs). Inverse variance fixed-effect meta-analysis was used to combine the genetic association for breast cancer risk across studies (Milne et al., 2017). In CIMBA, genotypes were generated by the Illumina OncoArray and imputed to the 1000 Genomes Project Phase 3 v5 EUR reference panel (Amos et al., 2016). A retrospective cohort analysis framework was adopted to estimate per-allele hazard ratios (HRs), modelling time-to-breast-cancer and stratified by country, Ashkenazi Jewish origin and birth cohort (Antoniou et al., 2005; Barnes et al., 2012). Fixed-effect meta-analysis (Willer, Li, & Abecasis, 2010) was performed to combine results across genotyping initiatives within the two consortia, assuming that the OR and HR estimates had roughly the same underlying relative risk. We restricted subsequent analyses to SNPs with an imputation *r*^2^ > .3, and an MAF > 0.005 across all platforms were included in the analysis (approximately 11.5 M).

For the ER+ subtype, meta-GWAS summary data based on 69,501 ER+ cases and 105,974 controls (part of the overall breast cancer samples) were included and analyzed (Mavaddat et al., 2015). For the ER− subtype, meta-GWAS summary data based on 21,468 ER− cases and 105,974 controls from the BCAC were combined with 9,414 additional *BRCA1* mutation-positive cases and 9,494 *BRCA1* mutation-positive controls from CIMBA (Milne et al., 2017).

To distinguish different genetic signals between ER+ and ER− subtypes, we further retrieved GWAS summary-level data on a case-only GWAS, which compared ER+ patients (sample size: 23,330 in iCOGs and 44,746 in OncoArray) to ER− patients (sample size: 5,479 and 11,856; Milne et al., 2017). Logistic regression was performed to test the association between genetic variants with known ER status in the two studies separately, adjusting for substudy and top PCs for iCOGs, and patients’ countries and top PCs for OncoArray. Results were then combined using a fixed-effect meta-analysis.

### 2.3 | Constructing expression weights

Before constructing the expression model (using GTEx data, regress gene expression on SNPs), we set several criteria to select eligible candidate genes for inclusion in the model (from the total 12,696 transcripts). We used a REML algorithm implemented in GCTA to estimate the *cis* (500 base-pair window surrounding transcription start site) SNP-heritability $\left(cis-h_{g}^{2}\right)$ for each transcript expression (Cai et al., 2014; J. Yang et al., 2010). Only genes with significant heritability (nominal *p*≤ .01) were included in the subsequent model construction (J. Yang, Lee, Goddard, & Visscher, 2011). The *p* values for null hypotheses $cis-h_{g}^{2}=0$ were computed using a likelihood ratio test. To account for population stratification, 20 PCs were always included as fixed effects. Consistent with previous research (ENCODE Project Consortium, 2012; J. Yang et al., 2010), we observed strong evidence for $cis-h_{g}^{2}$ on many genes (significantly non-zero for 1,355 genes).

We then constructed linear genetic predictors of gene expression for these genes. We performed five models: Bayesian Sparse Linear Mixed model, Best Linear Unbiased Predictor model, Elastic-net regression (with mixing parameter of 0.5), LASSO regression and Single best eQTL model. We used a fivefold cross-validation strategy to validate each model internally. Only genes with good model performance, corresponding to a prediction *r*^2^ value (the square of the correlation between predicted and observed expression) of at least 1% (0.10 correlation) in at least one of the five models, were included in subsequent TWAS analyses. The weights were chosen from the best performed model out of the five models. We adopted this additional filter to improve the interpretability and specificity of results: significant TWAS results based on models with little or no predictive ability likely result from pleiotropy or colocalization, not the effect of modeled gene’s expression levels. This additional filter narrowed the number of candidate genes to 901.

### 2.4 | Transcriptome-wide association study (TWAS) analyses

Using the functional weights of those 901 genes and summary level GWAS data, we assessed the association between predicted gene expression and breast cancer risk. We performed summary-based imputation using the ImpG-Summary algorithm (Pasaniuc et al., 2014). Briefly, let Z be a vector of standardized association statistics (*z* scores) of SNPs for a trait at a given *cis* locus, Σ*_s,s_* be the LD matrix from reference genotype data and let *W* = (*w*_1_*w*_2_*w*_3_… *w_j_*) be the weights from the expression prediction model precompiled using the reference panel. Under the null hypothesis that none of the SNPs with *w_i_* ≠ 0 is associated with disease, the test statistic *wz* /(*w*Σ*_s,s_w*′)^1/2^ follows a normal distribution with mean = 0 and variance = 1. To account for finite sample size and instances where Σ*_s,s_* was not invertible, we adjusted the diagonal of the matrix using a technique similar to ridge regression with *λ* = 0.1.

### 2.5 | Case-only TWAS

To assess whether genetically predicted expression was differentially associated with ER+ and ER− breast cancer, we applied the TWAS procedure described above to the *Z* statistics from the BCAC case-only analysis. Following arguments in Barfield et al. (2018) the standard TWAS statistic applied to a case-only GWAS results tests hypothesis *H*_0_: *β*_2_–*β*_1_ = 0. This is similar to a conventional multinomial logistic model for subtype-specific breast cancer risk, with expression log odds ratio *β*_2_ for ER− disease and *β*_1_ for ER+ disease, under which scenario, the expression log odds ratio comparing ER− to ER+ cases is *β*_2_–*β*_1_.

### 2.6 | Conditional analyses

Colocalization makes the interpretation of TWAS hits challenging (Mancuso et al., 2019; Wainberg et al., 2019). In addition to the main TWAS analysis, we also performed conditional and joint (COJO) multiple-SNP analysis at each TWAS significant gene location to distinguish colocalization, and to identify gene(s) independently responsible for the statistical association at each locus. COJO approximates the results of a joint conditional analysis including predicted expression levels from multiple proximal genes. The original COJO approach was designed to assess the association of individual SNPs with a phenotype; we used an extension that jointly models the associations between multiple linear combinations of individual SNPs (Gusev et al., 2016). We conducted two types of COJO: (a) For regions in which multiple associated features were identified (within 500 kb of each other, i.e., colocalization), we jointly modeled these significant TWAS genes to determine the strongest associated gene (or infer independent signals); (b) To provide information on whether the TWAS gene was responsible for the observed SNP-trait association, we also evaluated whether the GWAS-identified index SNPs remained significant after conditioning on the genes within the same region.

## 3 | RESULTS

### 3.1 | Breast cancer TWAS

We selected 12,696 transcripts from the 67 GTEx breast tissue samples of European-ancestry women that passed quality control. Based on GCTA-REML analysis, breast-tissue expression levels for 1,355 of these genes were heritable (*p* value for $cis-h_{g}^{2}<.01$). We then built linear predictors for these heritable genes and estimated prediction *r*^2^ using fivefold cross-validation. A total of 454 genes failed our cross-validation *r*^2^ requirement (*r*^2^ > .01), and we performed TWAS on the remaining 901 genes. We defined statistical significance for TWAS results as a marginal *p* < 5.5 × 10^−5^ (Bonferroni correction controlling the familywise error rate at ≤0.05 for the 901 genes).

First, to compare with previous GWAS findings and to demonstrate the validity of our results, we performed TWAS analysis in overall breast cancer. We identified 30 genes in 18 cytoband regions associated with breast cancer risk (Table 1). Of these regions, 11 (containing 21 genes) were previously reported breast cancer susceptibility loci (harboring one or more GWAS-significant SNP). Five genes in the remaining seven regions were previously reported in TWAS or EUGENE analyses (*LINC00886*, *CTD*-*2323K18.1*, *MAN2C1*, *NUP107*, and *CPNE1*), while the remaining four genes in these regions were novel (*MAEA*, *GDI2*, *ULK3*, and *HSD17B1P1*). *NUP107* and *CPNE1* did not pass a stringent Bonferroni significance threshold in Wu et al. (2018) but passed a less-tringent false discovery rate threshold.

We also carried out analyses focusing on breast cancer subtypes. We found 20 genes associated with ER+ breast cancer, and six genes associated with ER− breast cancer (*p* < .05/901 = 5.5 × 10^−5^; Table 1). In our results, all genes associated with ER− disease were also associated with ER+ disease, as well as with overall breast cancer risk. Using a more stringent threshold on the strength of the genetic predictor for expression (cross-validation *r*^2^ > .1; 383 genes passed this threshold), we found four TWAS significant (*p* < .05/383 = 1.3 × 10^−4^) genes for ER− disease, 14 genes for ER+ disease, and 19 genes for overall breast cancer (18 out of 19 genes are included in Table 1 except for one gene, *CTD/3110H11.1*). As before, these gene sets were nested within each other.

### 3.2 | Difference of TWAS signal across breast cancer subtypes

**FIGURE 1:**
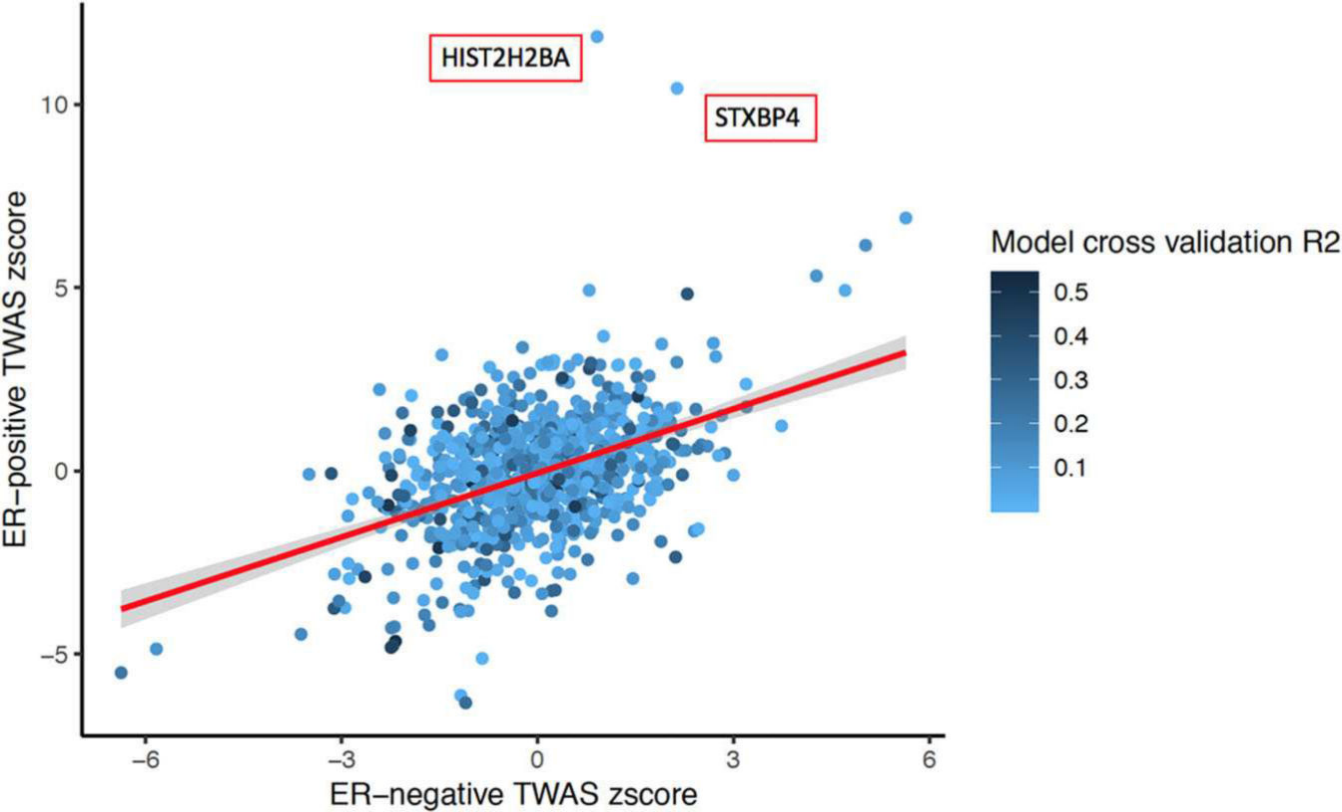
Scatter plot comparing the transcriptome-wide association study *z* scores in ER+ and ER− patients

We tested whether the imputed gene expression-breast cancer associations differed by subtype using GWAS summary statistics from a case-only analysis, which specifically compared ER+ with ER− breast cancer patients (see Section 2 for details), scanning through 901 eligible genes. Two genes, *HIST2H2BA* and *STXBP4,* showed significant associations (*p* < .05/901) with ER status among cases (Figure 1). These two genes were associated with ER+ breast cancer but not associated with ER− breast cancer.

### 3.3 | GWAS signal conditioning on TWAS gene expression

**FIGURE 2:**
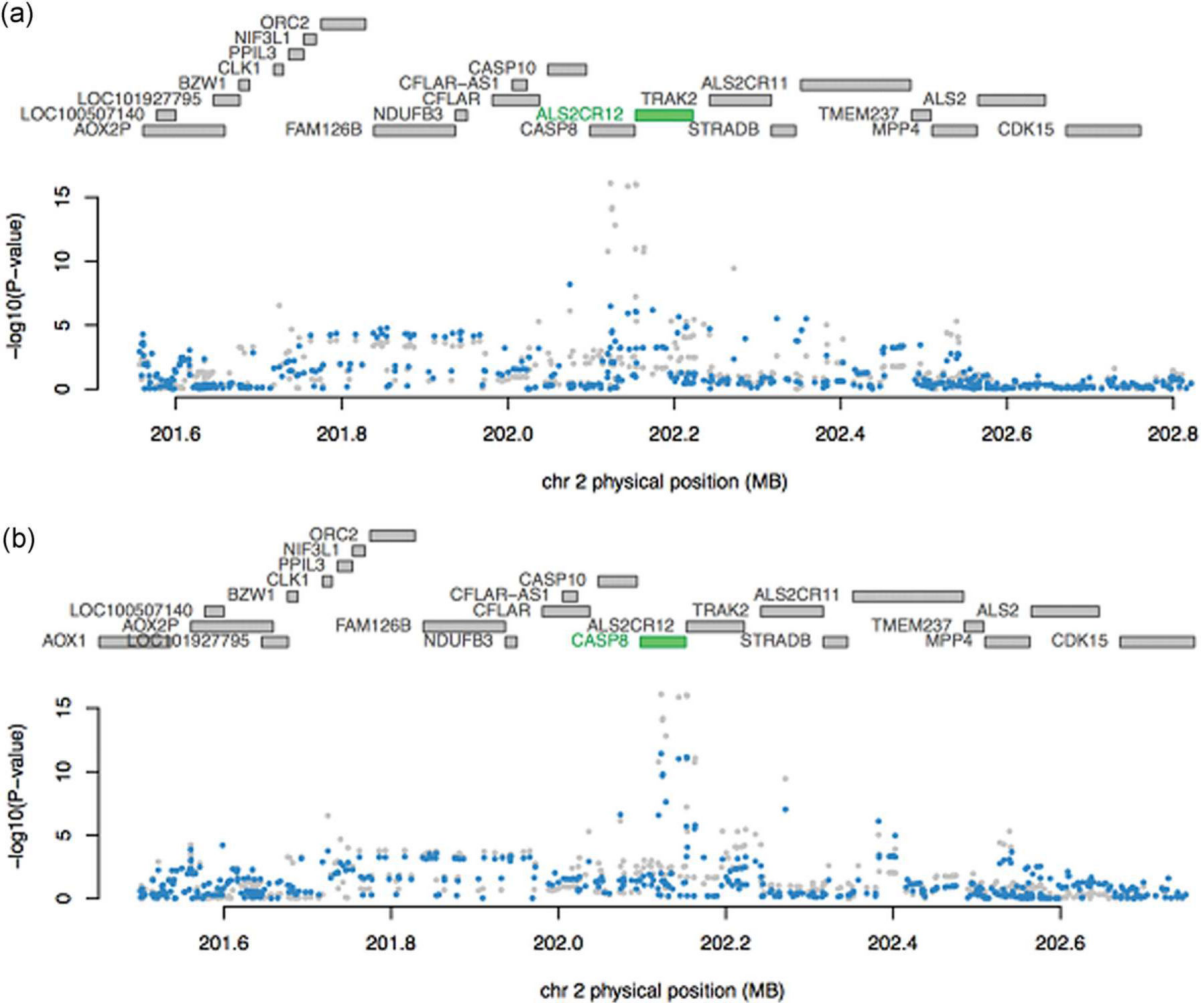
Conditional and joint analysis (COJO) for genes near a strong breast cancer GWAS hit. (a) COJO results adjusting for predicted expression of *ALS2CR12*. After conditioning on *ALS2CR12*, almost all original significant GWAS signals (grey dots) disappear (blue dots). (b) COJO results adjusting for the predicted expression of *CASP8*. After conditioning on *CASP8,* some of the original GWAS significant signals (grey dots) remains (blue dots)

As shown in Table 2, 21 (of 30) TWAS-significant genes were located near GWAS signals. To examine whether the observed GWAS signal within the gene region could be explained by the expression of that gene, we performed additional analyses conditioning SNP-cancer associations on the predicted expression of that particular significant TWAS gene (See Section 2 and Figure S1, for details). We found that for most regions, GWAS SNPs were no longer associated with the risk of breast cancer once conditioned on the expression of TWAS gene in the region: 15 of 21 genes had no SNPs with a conditional GWAS *p* value smaller than the genome-wide significant threshold (5 × 10^−8^). Thus, there were six genes for which the GWAS SNP remained significantly associated with breast cancer risk at the genome-wide threshold (5 × 10^−8^) after conditioning on TWAS gene expression. The region containing *HIST2H2BA* had only one genome-wide significant SNP remaining, and the region containing *ZNF155* and *ZNF404* had five genome-wide significant SNPs remaining, indicating that the expression of identified genes might explain some but not all of the SNP-breast cancer associations in these regions. For *CASP8* and *MRPL23*-*AS1* regions, half of the GWAS hits remained genome-wide significant, and for the *RP11*-*554A11.9* region, 33 out of 36 GWAS SNPs remained (Figures 2 and S1). These results suggest that the genetic association between breast cancer risk and those regions may not be mediated by transcriptional regulation of the genes on which we conditioned.

### 3.4 | Mutually adjusting for TWAS-significant genes in the same region

**FIGURE 3:**
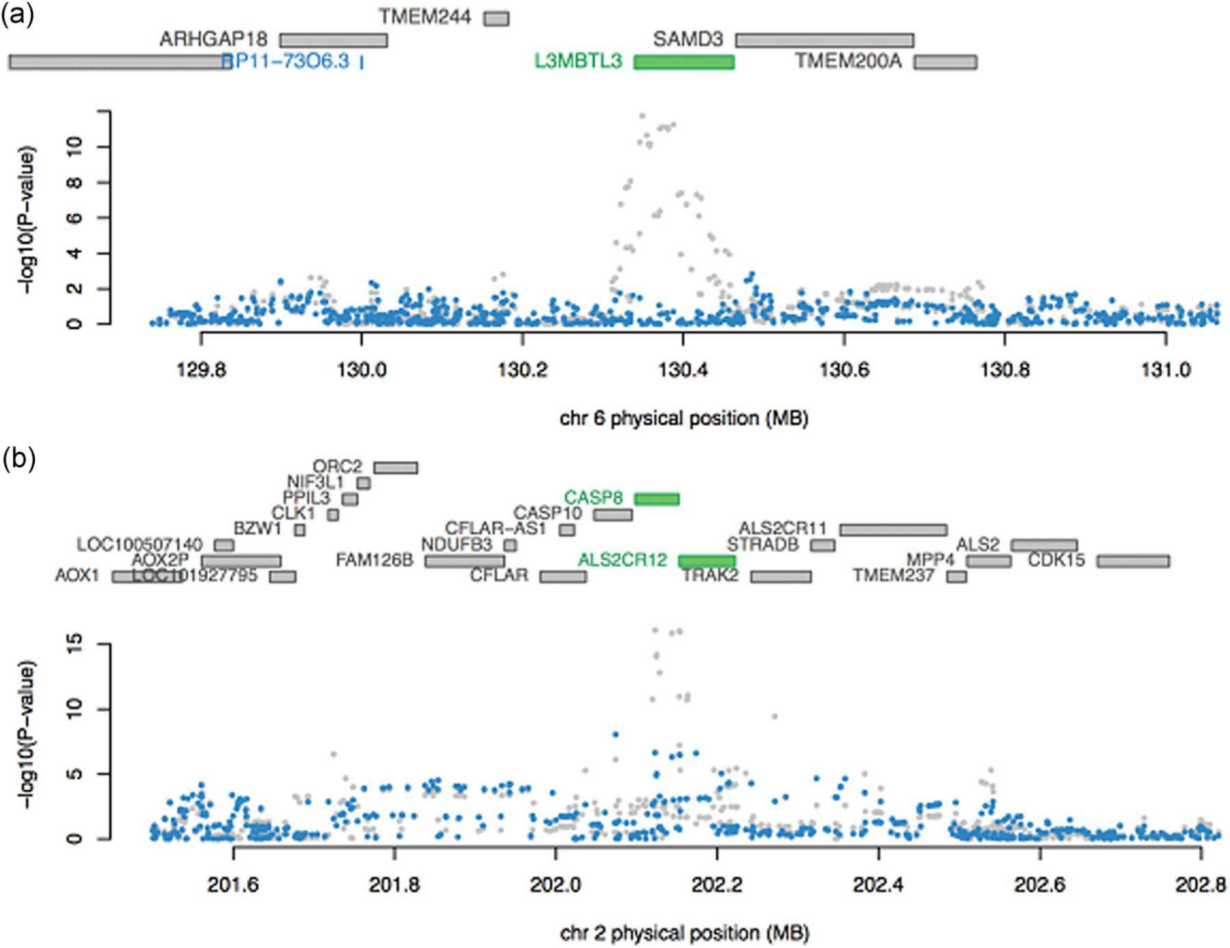
COJO for regions with multiple TWAS associations. For each plot, the top panel shows all genes in the locus. After COJO analysis, the marginally associated genes are highlighted in blue, while those that remain jointly significant are highlighted in green (in this case, *L3MBTL3*, *CASP8*, and *ALS2C12*). The bottom panel shows a Manhattan plot of the GWAS signals before (gray) and after (blue) conditioning on the significant (green) genes. (a) COJO results for 6q22 (only one gene remains significant after COJO). (b) COJO results for 2q33 (an example of multiple genes remaining jointly remain significant after COJO). COJO, conditional and joint analysis; GWAS, genome-wide association studies; TWAS, transcriptome-wide association studies

As shown in Table 3, we identified six regions with more than one TWAS-significant gene: 2q33 (*CASP8*, *ALS2CR12*), 5q14 (*ATG10*, *ATP6AP1L*), 6q22 (*RP11*-*73O6.3*, *L3MBTL3*), 15q24 (*ULK3*, *MAN2C1, CTD*-*2323K18.1*), 17q21 (*LRRC37A4p*, *CRHR1*-*IT1*, *CRHR1*, *KANSL1*-*AS1*, *LRRC37A*, *LRRC37A2*), and 19q13 (*ZNF404*, *ZNF155*, *RP11*-*15A1.7*). After mutually conditioning on the predicted expression of all significant genes in the same regions, ten genes remained nominally significant (*p* < 0.05). For some regions, only one gene remained, *that is ATG10* for 5q14, *L3MBTL3* for 6q22 and *CRHR1*-*IT1* for 17q21 (Figures 3a and S2); while for other regions, multiple genes remained significant, including *CASP8* and *ALS2CR12* for 2q33, *ULK3* and *MAN2C1* for 15q24, and *ZNF404, ZNF155*, and *RP11*-*15A1.7* for 19q13 (Figures 3b and S2).

## 4 | DISCUSSION

We conducted a TWAS analysis using GTEx mammary tissue gene expression data and GWAS summary data from the largest meta-analysis for breast cancer risk. We assessed associations between overall breast cancer risk and ER+ versus ER− disease. We found 30 genes significantly associated with overall breast cancer risk, 20 genes associated with the ER+ subtype, and six genes with the ER− subtype.

These results are consistent with previous reports from TWAS or similar gene-based approaches, which used various algorithms to build gene expression models. For example, of the 30 genes that we found significantly related to overall breast cancer risk, 23 were also significant in Wu et al. (2018) with very similar test statistics (correlation = 0.96 for the *z* scores between our and Wu’s results), and six were significant in Ferreira et al. (2019). One of the six genes we classified as significantly associated with ER− breast cancer was also found significantly associated with ER− breast cancer in Ferreira et al. (2019). Among these studies, the approach taken by Wu et al. was the most similar to ours. Only seven of the 30 genes that we identified were not identified by Wu et al. (2018), probably due to different *cis*-SNP selection criteria and different candidate genes selected for testing. We defined *cis*-SNPs using a 500 KB window around the gene boundary and included only candidate genes with a significant heritability, while Wu et al. used a 2 MB *cis*-SNP window and included genes with a prediction performance of at least 0.01 without heritability filtering. For genes whose expression could not be predicted well, Wu et al. built models using only SNPs located in promoter or enhancer regions. Despite these methodological differences, the two TWAS results were highly concordant. However, we did not replicate any of the findings in Hoffman et al. (2017) and Gao et al. (2017), which may reflect the smaller sample size of the breast cancer GWAS used in their analyses (3,370 cases and 19,717 controls in Hoffman et al.; 10,597 overall breast cancer cases, 3,879 ER− cases and 11,358 controls in Gao et al.). Specifically, three of the previously reported genes were excluded by our stringent QC procedure (*DHODH*, *ANKLE1* from Hoffman et al. and *TP53INP2* from Gao et al. were not heritable in our analysis) and one was not significant in our analysis (*RCCD1* from Hoffman et al. *p* = .0032 for overall breast cancer). Both Hoffman et al. and Gao et al. used GWAS results based on a mixed population of European, African, and Asian ancestry (which shared a small set of European samples with our GWAS: *N* < 5,700 individuals from CGEMS and the BPC3, less than 2% of our GWAS sample). They also used different tissues to build their prediction weights: overall breast tissue (men and women combined, all ethnicities) and whole blood tissue (men and women combined, European ancestry).

Of the 30 genes associated with breast cancer risk in our study, 21 fell into known GWAS regions whereas nine were not close to any known GWAS hit and were, therefore, considered novel. Of these nine genes, five were identified and discussed in Wu et al. (2018) or Ferreira et al. (2019). The four genes uniquely identified in the present study were *GDI2*, *HSD17B1P1*, *MAEA*, and *ULK3*, several of which have been reported to play a role in breast tumorigenesis or related biological processes. For example, the expression of *GDI2* has been linked with breast cancer through its contribution to enhanced epidermal growth factor receptor endocytosis (EGFR; de Graauw et al., 2014). *HSD17B1P1* is a pseudo-gene related to *HSD17*, which participates in steroid hormone biosynthesis, metabolism, and signaling pathways potentially related to breast cancer risk (Jakubowska et al., 2010). These findings lend support to our results and suggested that further investigation into the roles of the novel genes identified for breast cancer is required.

We performed several conditional analyses not reported in previous TWAS. We examined the local GWAS signals conditioning on the expression of TWAS genes, to provide a measure of how well the expression level of identified TWAS genes explained the local GWAS signals. For many loci, these genes explained a large proportion of the local GWAS signals and were thus candidates for downstream experimental validation. We also identified candidate genes driving the statistical associations in regions with more than one TWAS gene (usually also regions with known GWAS risk loci) by jointly modeling multiple nominally significant genes. For example, previous studies have suggested that polymorphisms in *CASP8* are associated with breast cancer risk (Cox et al., 2007), whereas a recent paper has shown that the most significant signal in this region is for the imputed intronic SNP rs1830298 in *ALS2CR12* (telomeric to *CASP8*; Lin et al., 2015). Our results provide clarification on whether *CASP8* or *ALS2CR12* expression were more strongly associated with breast cancer risk, since both genes remained significantly associated with breast cancer risk after conditioning on the expression of the other (the conditional *p* value for *ALS2CR12* was 3.70 × 10^−6^, whereas the conditional *p* value for *CASP8* was .05). Eleven of the 12 GWAS hits disappeared after adjusting for the expression of *ALS2CR12*, while half of the GWAS hits remained after adjusting for the expression of *CASP8*. Therefore, we believe that *ALS2CR12* SNPs have a stronger effect and are associated with breast cancer through *ALS2CR12* expression, while *CASP8* remains an additional independent hit, consistent with the latest fine-mapping results (Lin et al., 2015).

Because the genes found to be associated with ER− disease were also associated with ER+ disease, and these, in turn, were associated with overall breast cancer risk, it is difficult to conclude whether the differences in gene sets are due to distinct mechanisms underlying breast cancer subtypes or due to a lack of statistical power because of the smaller disease subtype sample sizes. To address this question, we further incorporated a case-only TWAS comparing ER+ versus ER− breast cancer. We identified two genes, *STXBP4* and *HIST2H2BA*, associated with ER status, which were significantly associated only with ER+ but not ER− breast cancer. Previous studies supported the link between rs6504950 (a SNP in *STXBP4*) and overall breast cancer risk (Antoniou et al., 2010; Warren Andersen et al., 2013). It has also been hypothesized that the risk allele for the two top breast cancer candidate SNPs, rs2787486 and rs244353, affected gene expression of *STXBP4* (Darabi et al., 2016) and CD4 memory cells (Hnisz et al., 2013). One potential explanation for the association between *STXBP4* and breast cancer risk is that it encodes syntaxin binding protein 4, a scaffold protein. In addition, *STXBP4* functions to stabilize and degrade TP63 isoform (a member of the TP53 tumor suppressor protein family), a biologically plausible candidate cancer susceptibility gene. Similarly, SNPs rs2580520 and rs11249433 upstream of *HIST2H2BA* have been identified as breast cancer susceptibility alleles in a previous GWAS (Bogdanova, Helbig, & Dörk, 2013). Our results suggest that functional and pathway analyses targeting these two genes are likely to shed new light on the differences in tumorigenesis and progression mechanisms between ER+ and ER− patients.

By building gene expression linear predictors in GTEx breast tissue, our analysis offers a tissue-specific model of gene expression. The gene regulatory mechanisms in female breast tissue are arguably the most suitable for studying breast cancer. Moreover, by restricting our reference population to women of European ancestry, rather than mixing genders and ancestries, the resulting gene expression model was a better match to our breast cancer GWAS summary statistics. By using the largest GWAS meta-analysis currently available, we greatly improved the power compared with previous work by Hoffman et al. (2017) and Gao et al. (2017). Finally, by using case-only GWAS summary statistics, we provided insights into genes associated with breast cancer subtype specific risk compared with Wu et al. (2018) and Ferreira et al. (2019).

Similar to previous work by Wu et al. (2018) and Hoffman et al. (2017), our analyses focused on genetic tools trained using expression from breast tissue, chosen because of its direct relevance to breast carcinogenesis. However, given the relatively small sample size in the breast tissue eQTL panel, this choice limited both our power to detect genes with *cis*-heritable expression and the precision of estimated genetic predictors for heritable transcripts. The genetic regulation of expression is constant across tissues for many genes, suggesting that considering other tissues with larger eQTL sample sizes or combining eQTL evidence across tissues may improve power. In addition, other tissues may be relevant for breast cancer development. For example, considering that obesity and hormonal signaling have been linked to breast cancer risk (Bertolini, 2013), gene expression in adipose tissue and brain tissue may have parallel involvement with breast cancer etiology. We are currently developing methods for cross-tissue TWAS, using sCCA (sparse canonical correlation analysis) to build features that combine gene expression values across tissues that share similar genetic regulation mechanisms, while allowing tissues with different regulation patterns to contribute to different features (Feng, Pasaniuc, Major, & Kraft, 2018).

In conclusion, we have identified new breast cancer target genes both for functional experiments and as causal gene candidates in the significant TWAS gene regions. We have also identified associations between gene expression and breast cancer risk specific to disease subtypes, where two novel genes have been found specifically associated with ER+ breast cancer risk. This analytic strategy warrants application in studies aimed at defining the genomic architecture of cancers other than breast cancer.

## Supplementary Material

supplementary material

## Figures and Tables

**TABLE 1:** Genes significantly associated with the overall breast cancer, estrogen receptor positive and negative subtypes, and estrogen receptor status, as identified by TWAS

Cytoband	Gene	Chromosome: Position (start–end)	Number of SNPs	Heritability	Cross validation *r*^2^	TWAS *p* values			
						Overall vs. controls	ER+ vs. controls	ER− vs. controls	ER+ vs. ER−
p11.2	HIST2H2BA	1: 120906028–120915073	77	0.10	0.04	**3.1E–30**	**2.0E–32**	3.6E–01	**1.4E–07**
q21.1	NUDT17	1: 145586804–145589439	110	0.07	0.03	**1.7E–09**	**8.8E–10**	2.4E–01	3.3E–03
q33.1	ALS2CR12	2: 202152994–202222121	363	0.35	0.03	**2.2E–11**	**8.4E–07**	**2.5E–06**	8.8E–01
	CASP8	2: 202098166–202152434	371	0.32	0.15	**1.8E–07**	**8.2E–06**	2.9E–04	7.6E–01
q25.31	LINC00886	3: 1156465135–156534851	452	0.17	0.04	**4.7E–05**	1.9E–04	3.2E–03	7.3E–01
p16.3	MAEA	4: 1283639–1333925	374	0.42	0.23	**3.9E–05**	1.6E–04	2.3E–01	2.3E–01
q14.2	ATG10	5: 81267844–81551958	520	0.44	0.26	**1.9E–10**	**2.4E–10**	2.7E–01	2.4E–02
	ATP6AP1L	5: 81575281–81682796	467	0.62	0.51	**2.3E–07**	**3.3E–06**	2.9E–02	2.3E–01
q14.1	RP11–250B2.5	6: 81176675–81178797	402	0.12	0.08	**6.9E–07**	7.3E–03	5.9E–03	8.2E–01
q22.33	RP11–73O6.3	6: 130454555–1130465515	557	0.33	0.11	**4.5E–12**	**1.2E–06**	**5.2E–09**	5.5E–02
	L3MBTL3	6: 130334844–130462594	609	0.31	0.23	**8.5E–14**	**3.6E–08**	**1.8E–10**	1.4E–01
p15.1	GDI2	10: 5807186–5884095	727	0.29	0.01	**4.8E–07**	**3.1E–07**	4.0E–01	2.1E–01
p15.5	MRPL23-AS1	11: 2004467–2011150	449	0.34	0.09	**7.4E–06**	1.4E–04	2.9E–01	2.2E–01
q13.2	RP11–554A11.9	11: 68923378–68927220	428	0.23	0.08	**1.5E–06**	4.9E–04	7.1E–03	6.9E–01
q15	NUP107	12: 69082742–69136785	538	0.27	0.05	**6.1E–06**	**8.2E–07**	4.3E–01	1.0E–01
q24.3	ULK3	15: 75128457–75135538	286	0.19	0.15	**3.9E–05**	**2.1E–05**	2.8E–02	5.2E–01
	MAN2C1	15: 75648133–75659986	226	0.37	0.34	**7.4E–07**	1.7E–04	1.8E–03	6.3E–01
	CTD-2323K18.1	15: 75819491–75893546	226	0.36	0.19	**7.5E–07**	3.9E–04	2.3E–03	3.1E–01
q21.31	HSD17B1P1	17: 40698782–40700724	245	0.09	0.07	**1.4E–06**	1.8E–03	6.4E–03	3.9E–01
q21.32	LRRC37A4P	17: 43578685–43623042	149	0.34	0.35	**3.1E–10**	**1.4E–06**	2.2E–02	8.0E–01
	CRHR1-IT1	17: 43697694–43725582	87	0.35	0.41	**2.9E–10**	**1.8E–06**	2.7E–02	8.2E–01
	CRHR1	17: 43699267–43913194	86	0.09	0.18	**4.8E–08**	**1.8E–05**	2.5E–02	7.6E–01
	KANSL1-AS1	17: 44270942–44274089	27	0.27	0.43	**3.4E–10**	**1.4E–06**	2.5E–02	7.6E–01
	LRRC37A	17: 44370099–44415160	75	0.27	0.20	**7.9E–08**	**2.5E–05**	9.7E–02	4.5E–01
	LRRC37A2	17: 44588877–44630815	130	0.37	0.18	**3.1E–07**	8.5E–05	8.4E–02	5.8E–01
q22	STXBP4	17: 53062975–53241646	609	0.20	0.01	**1.4E–25**	**1.6E–25**	3.3E–02	**1.5E–06**
q13.2	ZNF404	19: 44376515–44388203	445	0.31	0.06	**2.0E–13**	**5.1E–12**	**1.7E–08**	6.9E–01
	ZNF155	19: 44472014–44502477	486	0.39	0.11	**8.8E–09**	**1.0E–07**	**2.0E–05**	3.3E–01
	RP11–15A1.7	19: 44501048–44506988	477	0.29	0.14	**9.7E–12**	**7.3E–10**	**5.1E–07**	5.5E–01
q11.23	CPNE1	20: 34213953–34220170	299	0.25	0.20	**5.3E–05**	3.3E–04	1.4E–01	4.9E–01

*Note*: Significant associations after Bonferroni adjustment (*p* < .05/901) are in bold.

Abbreviations: ER, estrogen receptor; SNP, single nucleotide polymorphisms; TWAS, transcriptome-wide association studies.

**TABLE 2:** Summary of conditional analysis at known breast cancer risk region

	Before conditional analysis			After conditional analysis				
Gene	Number of SNPs	Number of significant SNPs	Index GWAS SNP *p* value	Number of significant SNPs	Index SNP	Smallest conditional *p* values	Ratio^a^	Magnitude of change in the minimum *p* value before and after COJO
ALS2CR12	480	12	8.2E–17	1	rs3769823	6.40E–09	0.92	1.28E–08
ATG10	619	24	6.9E–13	0	rs891159	1.20E–07	1.00	5.75E–06
ATP6AP1L	581	24	6.9E–13	0	rs891159	1.70E–07	1.00	4.06E–06
CASP8	493	12	8.2E–17	6	rs3769823	3.90E–12	0.50	2.10E–05
CRHR1	229	13	1.5E–10	0	rs17763086	1.40E–05	1.00	1.07E–05
CRHR1-IT1	230	13	1.5E–10	0	rs17763086	9.90E–05	1.00	1.52E–06
HIST2H2BA	202	19	3.5E–52	1	rs11249433	7.40E–24	0.95	4.73E–29
KANSL1-AS1	34	13	1.5E–10	0	rs17763086	1.60E–01	1.00	9.38E–10
L3MBTL3	724	13	1.7E–12	0	rs6569648	1.40E–03	1.00	1.21E–09
LRRC37A	285	13	1.5E–10	0	rs17763086	4.60E–05	1.00	3.26E–06
LRRC37A4P	285	13	1.5E–10	0	rs17763086	4.60E–05	1.00	3.26E–06
MRPL23-AS1	557	36	2.4E–33	18	rs569550	1.20E–29	0.50	2.00E–04
NUDT17	112	17	1.5E–10	0	rs36107432	3.90E–05	1.00	3.85E–06
RP11-15A1.7	594	32	1E–16	0	rs10426528	4.60E–07	1.00	2.17E–10
RP11-250B2.5	503	8	2.7E–09	0	rs9343989	1.00E–03	1.00	2.70E–06
RP11-554A11.9	532	36	2.8E–44	33	rs680618	2.80E–44	0.08	1.00E+00
RP11-73O6.3	665	13	1.7E–12	0	rs6569648	1.70E–03	1.00	1.00E–09
STXBP4	687	46	2E–28	0	rs244353	2.10E–04	1.00	9.52E–25
ZNF155	597	32	1E–16	5	rs10426528	2.00E–09	0.84	5.00E–08
ZNF404	551	32	1E–16	0	rs10426528	5.30E–07	1.00	1.89E–10
LRRC37A2	152	2	2E–08	0	rs199498	1.80E–02	1.00	1.11E–06

Abbreviations: COJO, conditional and joint; GWAS, genome-wide association studies; SNP, single nucleotide polymorphisms.

a Proportion of marginally significant SNPs that are not significant in conditional analyses. Analysis was performed using GWAS summary statistics of ER+ subtypes. The difference between marginal SNP tests for association (GWAS p values) and the SNP p values conditional on significant TWAS genes provides some evidence regarding the independence of the TWAS and single-SNP association signals. The number and proportion of SNPs that are genome-wide significant before and after conditioning on a TWAS-significant gene summarizes the degree single-SNP associations are dependent on (or independent of) the TWAS association.

**TABLE 3:** Conditional and joint analysis of gene region with multiple TWAS significant genes

		Marginal TWAS		COJO	
Region^a^	Gene (colocalized)	*Z* score	*p* Value	*Z* score	*p* Value
2q33	**ALS2CR12**	6.7	2.15E–11	4.6	3.70E–06
	**CASP8**	−5.22	1.76E–07	−2	5.00E–02
5q14	**ATG10**	−6.37	1.85E–10	−6.37	1.85E–10
	ATP6AP1L	−5.18	2.25E–07	−0.85	0.4
6q22	RP11-73O6.3	−6.92	4.46E–12	0.18	0.86
	**L3MBTL3**	−7.46	8.45E–14	−7.46	8.45E–14
15q24	**ULK3**	−4.11	3.87E–05	−4.1	3.90E–05
	**MAN2C1**	−4.95	7.37E–07	−5	7.40E–07
	CTD-2323K18.1	−4.95	7.49E–07	−1.7	0.083
17q21	LRRC37A4P	6.29	3.12E–10	0.25	0.8
	**CRHR1-IT1**	−6.3	2.91E–10	−6.3	2.91E–10
	CRHR1	−5.46	4.84E–08	−0.28	0.78
	KANSL1-AS1	−6.28	3.37E–10	−0.04	0.97
	LRRC37A	−5.37	7.89E–08	1.83	0.07
	LRRC37A2	−5.12	3.07E–07	1.81	0.07
19q13	**ZNF404**	7.35	2.04E–13	3.5	0.001
	**ZNF155**	5.75	8.81E–09	−2	0.042
	**RP11-15A1.7**	6.81	9.67E–12	2.8	0.005

Abbreviations: COJO, conditional and joint; TWAS, transcriptome-wide association studies.

a Bolded genes remain significant in conditional analyses. Analysis was performed using GWAS summary statistics of ER+ subtypes. Our primary goal in these analyses is to establish whether any of the marginally significant TWAS genes remains significant after conditioning for the most significant gene in the region; sincesince all of the regions with multiple significant genes contain 2–3 significant genes, using a conditional *p* value threshold of .05 is a reasonable threshold for identifying independent signals.

## Data Availability

The GWAS summary data that support the findings of overall, ER−, ER+ breast cancer results are openly available in the Breast Cancer Association Consortium (BCAC). The GWAS summary data that support the findings of case-only breast cancer results will also be made available at the BCAC website. http://bcac.ccge.medschl.cam.ac.uk/bcacdata/oncoarray/gwas-icogs-and-oncoarray-summary-results/ (Michailidou et al., 2017, pp. 92–94).

## References

[R1] Amos CI, Dennis J, Wang Z, Byun J, Schumacher FR, Gayther SA, … Easton DF (2016). The OncoArray Consortium: A network for understanding the genetic architecture of common cancers. Cancer Epidemiology, Biomarkers & Prevention, 10.1158/1055-9965.EPI-16-0106 PMC5224974 27697780

[R2] Antoniou AC, Beesley J, McGuffog L, Sinilnikova OM, Healey S, Neuhausen SL, … Cimba (2010). Common breast cancer susceptibility alleles and the risk of breast cancer for BRCA1 and BRCA2 mutation carriers: Implications for risk prediction. Cancer Research, 70(23), 9742–9754. 10.1158/0008-5472.CAN-10-1907 21118973 PMC2999830

[R3] Antoniou AC, Goldgar DE, Andrieu N, Chang-Claude J, Brohet R, Rookus MA, & Easton DF (2005). A weighted cohort approach for analysing factors modifying disease risks in carriers of high-risk susceptibility genes. Genetic Epidemiology, 29(1), 1–11. 10.1002/gepi.20074 15880399

[R4] Atchley DP, Albarracin CT, Lopez A, Valero V, Amos CI, Gonzalez-Angulo AM, … Arun BK (2008). Clinical and pathologic characteristics of patients with BRCA-positive and BRCA-negative breast cancer. Journal of Clinical Oncology, 26(26), 4282–4288. 10.1200/JCO.2008.16.6231 18779615 PMC6366335

[R5] Barfield R, Feng H, Gusev A, Wu L, Zheng W, Pasaniuc B, & Kraft P (2018). Transcriptome-wide association studies accounting for colocalization using Egger regression. Genetic Epidemiology, 42(5), 418–433. 10.1002/gepi.22131 29808603 PMC6342197

[R6] Barnes DR, Lee A, EMBRACE Investigators, kConFab Investigators, Easton DF, & Antoniou AC (2012). Evaluation of association methods for analysing modifiers of disease risk in carriers of high-risk mutations. Genetic Epidemiology, 36(3), 274–291. 10.1002/gepi.21620 22714938

[R7] Beggs AD, & Hodgson SV (2009). Genomics and breast cancer: The different levels of inherited susceptibility. European Journal of Human Genetics, 17(7), 855–856. 10.1038/ejhg.2008.235 19092772 PMC2986490

[R8] Bertolini F (2013). Adipose tissue and breast cancer progression: A link between metabolism and cancer. Breast, 22(Suppl 2), S48–S49. 10.1016/j.breast.2013.07.009 24074792

[R9] Blows FM, Driver KE, Schmidt MK, Broeks A, van Leeuwen FE, Wesseling J, … Huntsman D (2010). Subtyping of breast cancer by immunohistochemistry to investigate a relationship between subtype and short and long term survival: A collaborative analysis of data for 10,159 cases from 12 studies. PLOS Medicine, 7(5):e1000279. 10.1371/journal.pmed.1000279 20520800 PMC2876119

[R10] Bogdanova N, Helbig S, & Dörk T (2013). Hereditary breast cancer: Ever more pieces to the polygenic puzzle. Hereditary Cancer in Clinical Practice, 11(1), 12. 10.1186/1897-4287-11-12 24025454 PMC3851033

[R11] Bray F, Ferlay J, Soerjomataram I, Siegel RL, Torre LA, & Jemal A (2018). Global cancer statistics 2018: GLOBOCAN estimates of incidence and mortality worldwide for 36 cancers in 185 countries. CA: A Cancer Journal for Clinicians, 68(6), 394–424. 10.3322/caac.21492 30207593

[R12] Cai Q, Zhang B, Sung H, Low S-K, Kweon S-S, Lu W, … Zheng W (2014). Genome-wide association analysis in East Asians identifies breast cancer susceptibility loci at 1q32.1, 5q14.3 and 15q26.1. Nature Genetics, 46(8), 886–890. 10.1038/ng.3041 25038754 PMC4127632

[R13] Cox A, Dunning AM, Garcia-Closas M, Balasubramanian S, Reed MWR, Pooley KA, … Johnson N, Breast Cancer Association Consortium. (2007). A common coding variant in CASP8 is associated with breast cancer risk. Nature Genetics, 39(3), 352–358. 10.1038/ng1981 17293864

[R14] Darabi H, Beesley J, Droit A, Kar S, Nord S, Moradi Marjaneh M, … Dunning AM (2016). Fine scale mapping of the 17q22 breast cancer locus using dense SNPs, genotyped within the Collaborative Oncological Gene-Environment Study (COGs). Scientific Reports, 6, 32512. 10.1038/srep32512 27600471 PMC5013272

[R15] Delaneau O, Marchini J, & Zagury J-F (2011). A linear complexity phasing method for thousands of genomes. Nature Methods, 9(2), 179–181. 10.1038/nmeth.1785 22138821

[R16] Dunning AM, Michailidou K, Kuchenbaecker KB, Thompson D, French JD, Beesley J, … Edwards SL (2016). Breast cancer risk variants at 6q25 display different phenotype associations and regulate ESR1, RMND1 and CCDC170. Nature Genetics, 48(4), 374–386. 10.1038/ng.3521 26928228 PMC4938803

[R17] Easton DF, Pooley KA, Dunning AM, Pharoah PDP, Thompson D, Ballinger DG, … Ponder BAJ (2007). Genome-wide association study identifies novel breast cancer susceptibility loci. Nature, 447(7148), 1087–1093. 10.1038/nature05887 17529967 PMC2714974

[R18] ENCODE Project Consortium. (2012). An integrated encyclopedia of DNA elements in the human genome. Nature, 489(7414), 57–74. 10.1038/nature11247 22955616 PMC3439153

[R19] Feng H, Pasaniuc B, Major M, & Kraft P (2018). Sparse canonical correlation analysis (sCCA) significantly improves power of cross-tissue transcriptome-wide association studies (TWAS). Genetic Epidemiology, 42, 698.

[R20] Ferreira MA, Gamazon ER, Al-Ejeh F, Aittomäki K, Andrulis IL, Anton-Culver H, … Chenevix-Trench G (2019). Genome-wide association and transcriptome studies identify target genes and risk loci for breast cancer. Nature Communications, 10(1), 1741. 10.1038/s41467-018-08053-5 PMC6465407 30988301

[R21] Gamazon ER, Wheeler HE, Shah KP, Mozaffari SV, Aquino-Michaels K, Carroll RJ, … Im HK (2015). A gene-based association method for mapping traits using reference transcriptome data. Nature Genetics, 47(9), 1091–1098. 10.1038/ng.3367 26258848 PMC4552594

[R22] Gao G, Pierce BL, Olopade OI, Im HK, & Huo D (2017). Trans-ethnic predicted expression genome-wide association analysis identifies a gene for estrogen receptor-negative breast cancer. PLOS Genetics, 13(9):e1006727. 10.1371/journal.pgen.1006727 28957356 PMC5619687

[R23] Garcia-Closas M, Couch FJ, Lindstrom S, Michailidou K, Schmidt MK, Brook MN, … Kraft P (2013). Genome-wide association studies identify four ER negative-specific breast cancer risk loci. Nature Genetics, 45(4), 392–398, 398e1–2. 10.1038/ng.2561 23535733 PMC3771695

[R24] de Graauw M, Cao L, Winkel L, van Miltenburg M. H. a M., le Dévédec SE, Klop M, … van de Water B (2014). Annexin A2 depletion delays EGFR endocytic trafficking via cofilin activation and enhances EGFR signaling and metastasis formation. Oncogene, 33(20), 2610–2619. 10.1038/onc.2013.219 23792445

[R25] GTEx Consortium. (2015). Human genomics. The Genotype-Tissue Expression (GTEx) pilot analysis: Multitissue gene regulation in humans. Science, 348(6235), 648–660. 10.1126/science.1262110 25954001 PMC4547484

[R26] Gusev A, Ko A, Shi H, Bhatia G, Chung W, Penninx BWJH, … Pasaniuc B (2016). Integrative approaches for large-scale transcriptome-wide association studies. Nature Genetics, 48(3), 245–252. 10.1038/ng.3506 26854917 PMC4767558

[R27] Hnisz D, Abraham BJ, Lee TI, Lau A, Saint-André V, Sigova AA, … Young RA (2013). Super-enhancers in the control of cell identity and disease. Cell, 155(4), 934–947. 10.1016/j.cell.2013.09.053 24119843 PMC3841062

[R28] Hoffman JD, Graff RE, Emami NC, Tai CG, Passarelli MN, Hu D, … Witte JS (2017). Cis-eQTL-based trans-ethnic meta-analysis reveals novel genes associated with breast cancer risk. PLOS Genetics, 13(3):e1006690. 10.1371/journal.pgen.1006690 28362817 PMC5391966

[R29] Howie BN, Donnelly P, & Marchini J (2009). A flexible and accurate genotype imputation method for the next generation of genome-wide association studies. PLOS Genetics, 5(6): e1000529. 10.1371/journal.pgen.1000529 19543373 PMC2689936

[R30] Jakubowska A, Gronwald J, Menkiszak J, Górski B, Huzarski T, Byrski T, … Hamann U (2010). BRCA1associated breast and ovarian cancer risks in Poland: No association with commonly studied polymorphisms. Breast Cancer Research and Treatment, 119(1), 201–211. 10.1007/s10549-009-0390-5 19360465

[R31] Lin W-Y, Camp NJ, Ghoussaini M, Beesley J, Michailidou K, Hopper JL, … Cox A (2015). Identification and characterization of novel associations in the CASP8/ALS2CR12 region on chromosome 2 with breast cancer risk. Human Molecular Genetics, 24(1), 285–298. 10.1093/hmg/ddu431 25168388 PMC4334820

[R32] Mancuso N, Freund MK, Johnson R, Shi H, Kichaev G, Gusev A, & Pasaniuc B (2019). Probabilistic fine-mapping of transcriptome-wide association studies. Nature Genetics, 51(4), 675–682. 10.1038/s41588-019-0367-1 30926970 PMC6619422

[R33] Mancuso N, Shi H, Goddard P, Kichaev G, Gusev A, & Pasaniuc B (2017). Integrating gene expression with summary association statistics to identify genes associated with 30 complex traits. American Journal of Human Genetics, 100(3), 473–487. 10.1016/j.ajhg.2017.01.031 28238358 PMC5339290

[R34] Mavaddat N, Antoniou AC, Easton DF, & Garcia-Closas M (2010). Genetic susceptibility to breast cancer. Molecular oncology, 4(3), 174–191. 10.1016/j.molonc.2010.04.011 20542480 PMC5527934

[R35] Mavaddat N, Pharoah PDP, Michailidou K, Tyrer J, Brook MN, Bolla MK, … Garcia-Closas M (2015). Prediction of breast cancer risk based on profiling with common genetic variants. Journal of the National Cancer Institute, 107(5), 10.1093/jnci/djv036 PMC4754625 25855707

[R36] McCarthy S, Das S, Kretzschmar W, Delaneau O, Wood AR, Teumer A, … Mahajan A, Haplotype Reference Consortium. (2016). A reference panel of 64,976 haplotypes for genotype imputation. Nature Genetics, 48(10), 1279–1283. 10.1038/ng.3643 27548312 PMC5388176

[R37] Michailidou K, Beesley J, Lindstrom S, Canisius S, Dennis J, Lush MJ, … Easton DF (2015). Genome-wide association analysis of more than 120,000 individuals identifies 15 new susceptibility loci for breast cancer. Nature Genetics, 47(4), 373–380. 10.1038/ng.3242 25751625 PMC4549775

[R38] Michailidou K, Lindström S, Dennis J, Beesley J, Hui S, Kar S, … Easton DF (2017). Association analysis identifies 65 new breast cancer risk loci. Nature, 551(7678), 92–94. 10.1038/nature24284 29059683 PMC5798588

[R39] Milne RL, Kuchenbaecker KB, Michailidou K, Beesley J, Kar S, Lindström S, … Simard J (2017). Identification of ten variants associated with risk of estrogen-receptor-negative breast cancer. Nature Genetics, 49(12), 1767–1778. 10.1038/ng.3785 29058716 PMC5808456

[R40] Nicolae DL, Gamazon E, Zhang W, Duan S, Dolan ME, & Cox NJ (2010). Trait-associated SNPs are more likely to be eQTLs: Annotation to enhance discovery from GWAS. PLOS Genetics, 6(4):e1000888. 10.1371/journal.pgen.1000888 20369019 PMC2848547

[R41] Pasaniuc B, Zaitlen N, Shi H, Bhatia G, Gusev A, Pickrell J, … Price AL (2014). Fast and accurate imputation of summary statistics enhances evidence of functional enrichment. Bioinformatics, 30(20), 2906–2914. 10.1093/bioinformatics/btu416 24990607 PMC4184260

[R42] Seal S, Thompson D, Renwick A, Elliott A, Kelly P, Barfoot R, … Rahman N (2006). Truncating mutations in the Fanconi anemia J gene BRIP1 are low-penetrance breast cancer susceptibility alleles. Nature Genetics, 38(11), 1239–1241. 10.1038/ng1902 17033622

[R43] Turnbull C, Ahmed S, Morrison J, Pernet D, Renwick A, Maranian M, … Easton DF (2010). Genome-wide association study identifies five new breast cancer susceptibility loci. Nature Genetics, 42(6), 504–507. 10.1038/ng.586 20453838 PMC3632836

[R44] Wainberg M, Sinnott-Armstrong N, Mancuso N, Barbeira AN, Knowles DA, Golan D, … Kundaje A (2019). Opportunities and challenges for transcriptome-wide association studies. Nature Genetics, 51(4), 592–599. 10.1038/s41588-019-0385-z 30926968 PMC6777347

[R45] Warren Andersen S, Trentham-Dietz A, Gangnon RE, Hampton JM, Figueroa JD, Skinner HG, … Newcomb PA (2013). The associations between a polygenic score, reproductive and menstrual risk factors and breast cancer risk. Breast Cancer Research and Treatment, 140(2), 427–434. 10.1007/s10549-013-2646-3 23893088 PMC3799826

[R46] Willer CJ, Li Y, & Abecasis GR (2010). METAL: Fast and efficient meta-analysis of genomewide association scans. Bioinformatics, 26(17), 2190–2191. 10.1093/bioinformatics/btq340 20616382 PMC2922887

[R47] Wu L, Shi W, Long J, Guo X, Michailidou K, Beesley J, … Zheng W (2018). A transcriptome-wide association study of 229,000 women identifies new candidate susceptibility genes for breast cancer. Nature Genetics, 50(7), 968–978. 10.1038/s41588-018-0132-x 29915430 PMC6314198

[R48] Yang J, Benyamin B, McEvoy BP, Gordon S, Henders AK, Nyholt DR, … Visscher PM (2010). Common SNPs explain a large proportion of the heritability for human height. Nature Genetics, 42(7), 565–569. 10.1038/ng.608 20562875 PMC3232052

[R49] Yang J, Lee SH, Goddard ME, & Visscher PM (2011). GCTA: A tool for genome-wide complex trait analysis. American Journal of Human Genetics, 88(1), 76–82. 10.1016/j.ajhg.2010.11.011 21167468 PMC3014363

[R50] Yang XR, Chang-Claude J, Goode EL, Couch FJ, Nevanlinna H, Milne RL, … Garcia-Closas M (2011). Associations of breast cancer risk factors with tumor subtypes: A pooled analysis from the Breast Cancer Association Consortium studies. Journal of the National Cancer Institute, 103(3), 250–263. 10.1093/jnci/djq526 21191117 PMC3107570

[R51] Yovel Y, Franz MO, Stilz P, & Schnitzler H-U (2008). Plant classification from bat-like echolocation signals. PLOS Computational Biology, 4(3):e1000032. 10.1371/journal.pcbi.1000032 18369425 PMC2267002

