# Supplementary material for: Transcriptome-wide association study of breast cancer risk by estrogen-receptor status
Source: Genet Epidemiol. Author manuscript; Available in PMC 2021 Mar 23. (PMC7987299; doi:10.1002/gepi.22288)

A TRANSCRIPTOME-WIDE ASSOCIATION STUDY OF BREAST CANCER BY ESTROGEN RECEPTOR STATUS

Feng *et al.*

Supplementary Figure 1. Conditional analysis on gene with known GWAS hit.

1. ALS2CR12


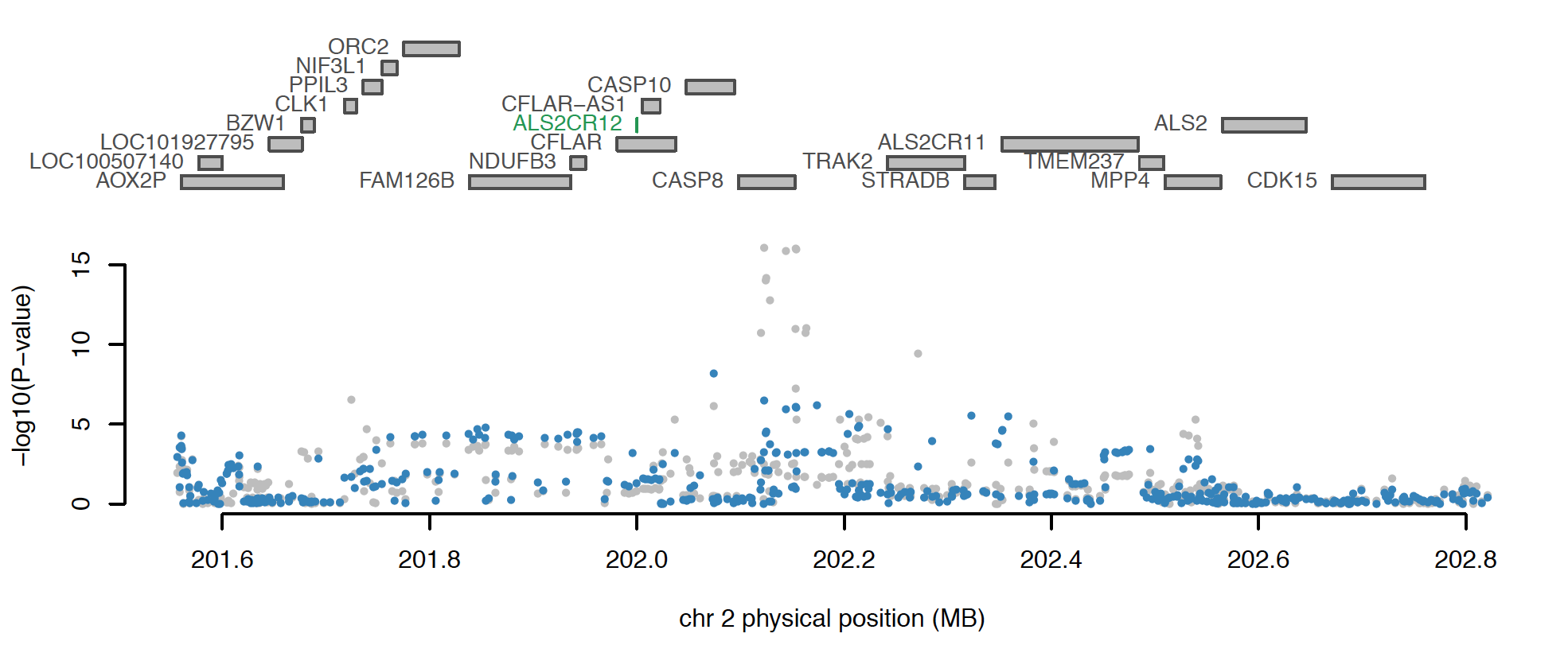


1. ATG10


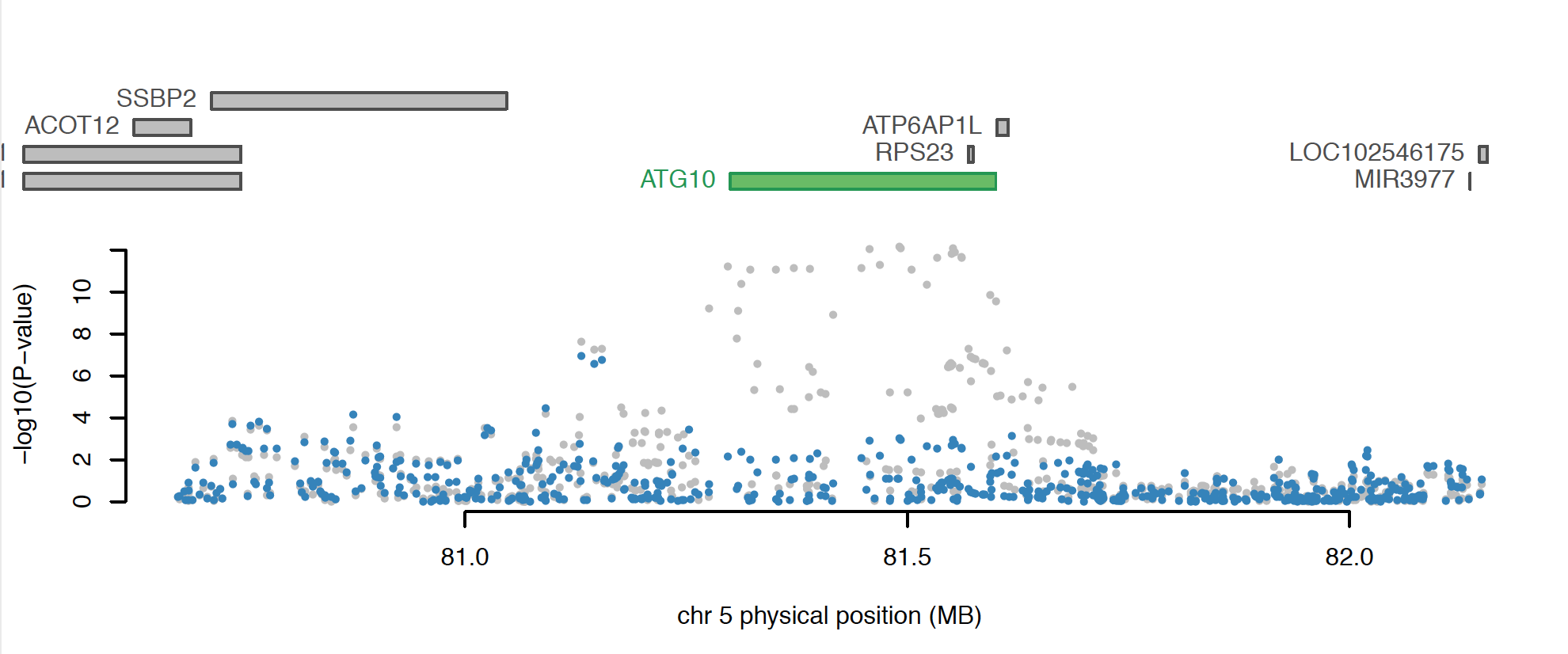


1. ATP6AP1L


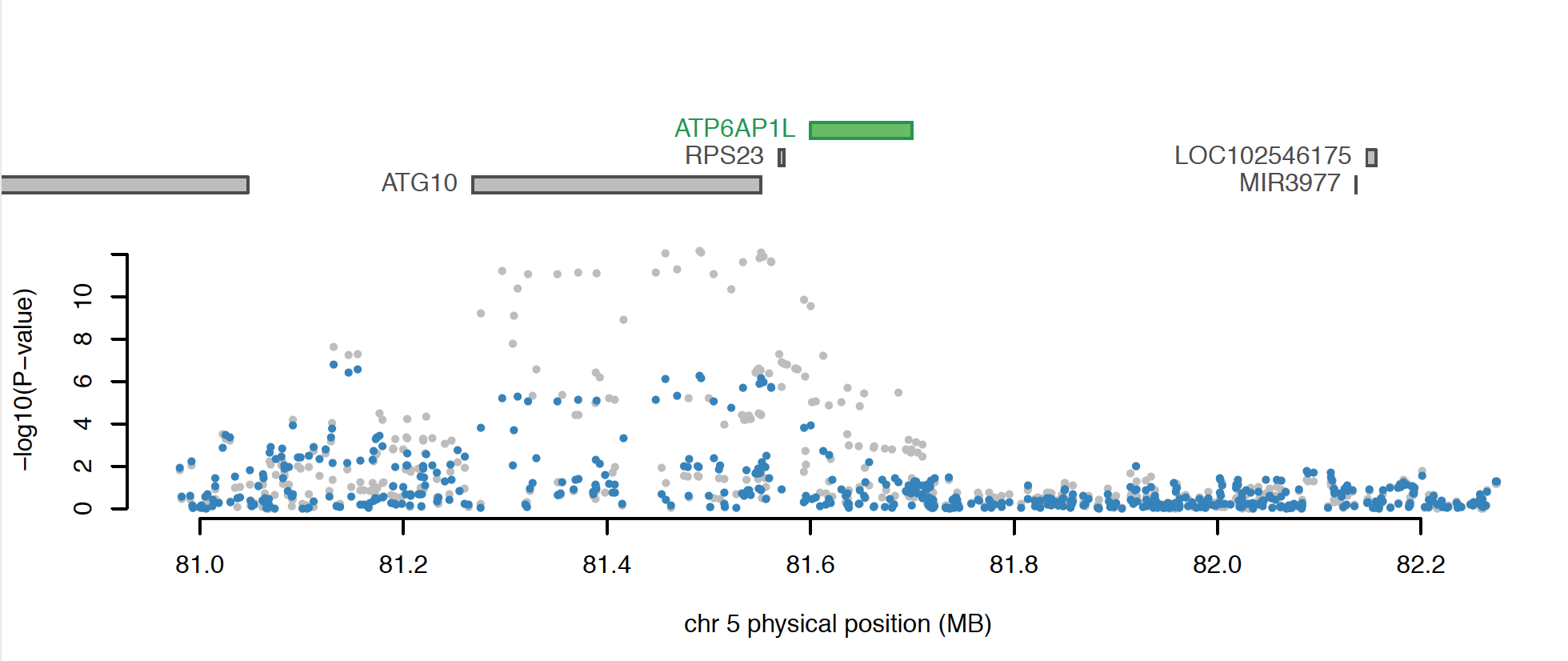


1. CASP8


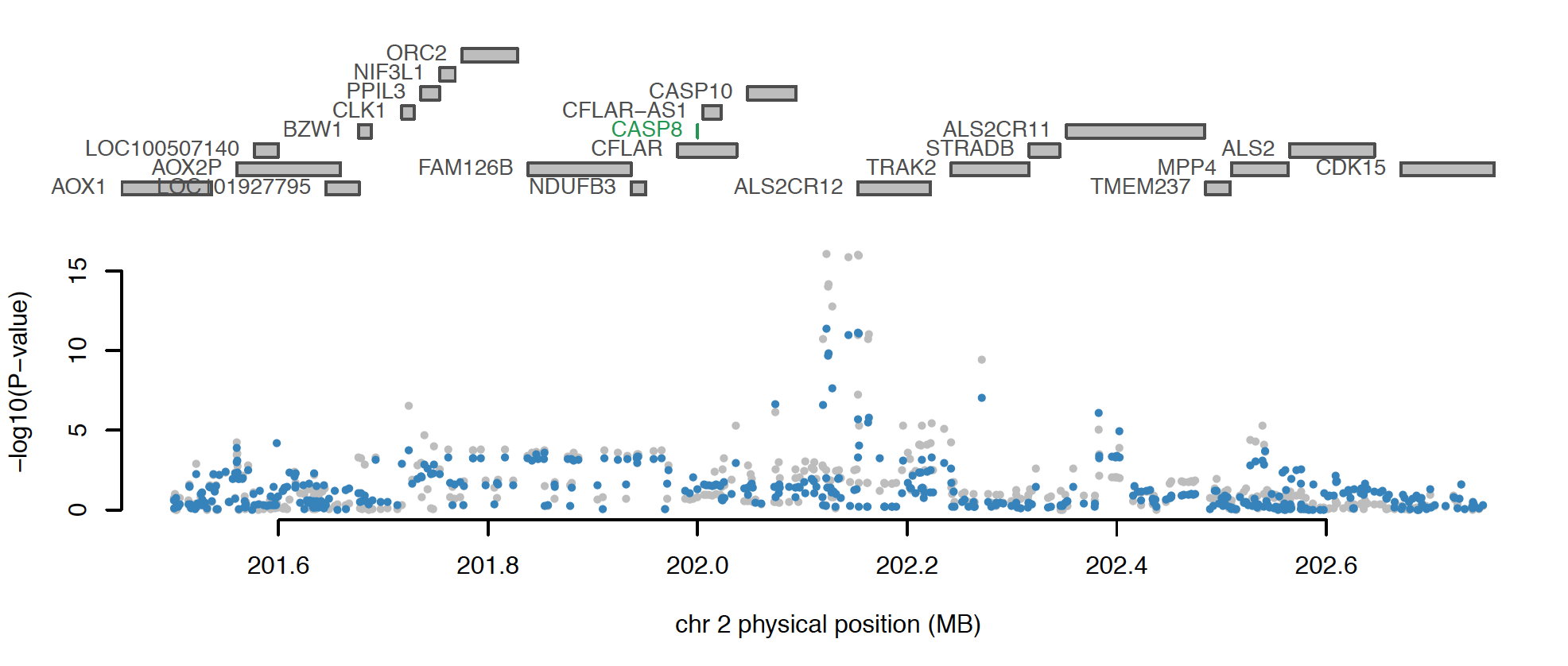


1. CRHR1-IT1


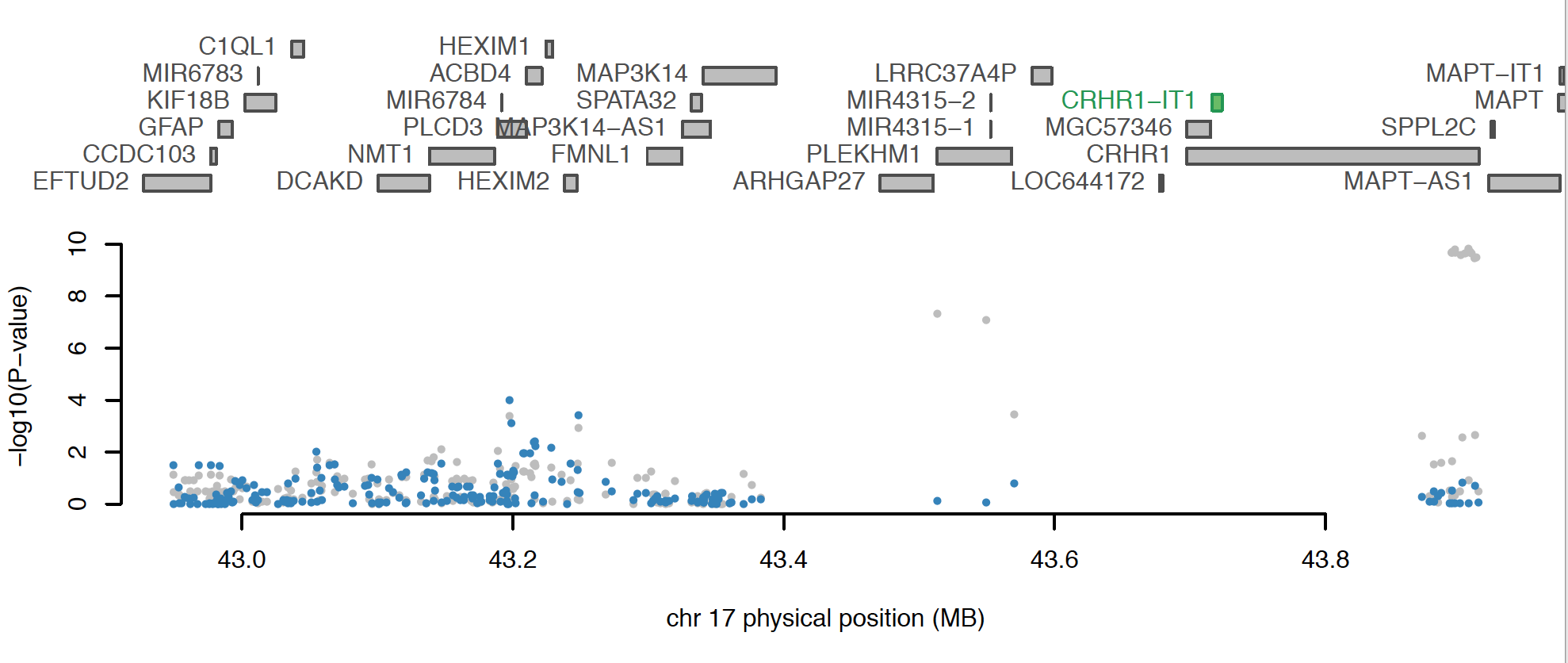


1. CRHR1


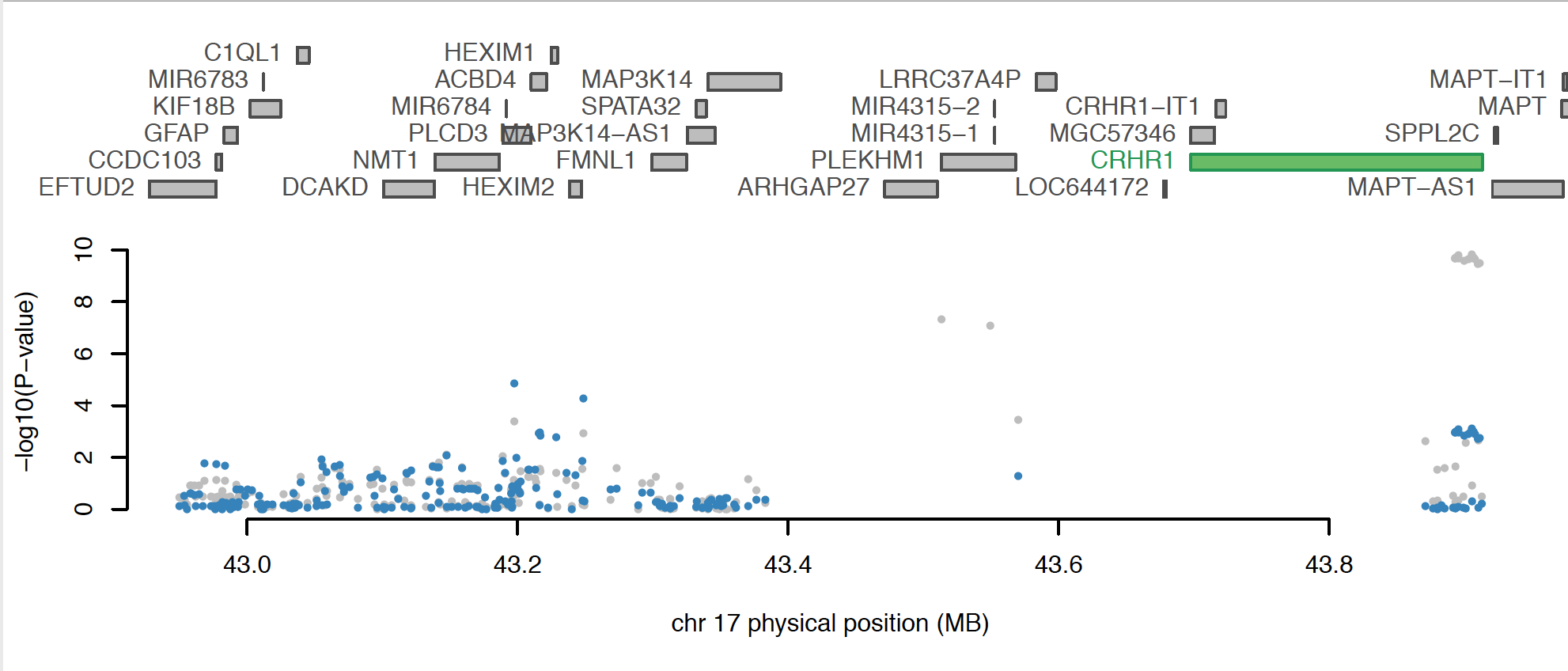


1. HIST2H2BA


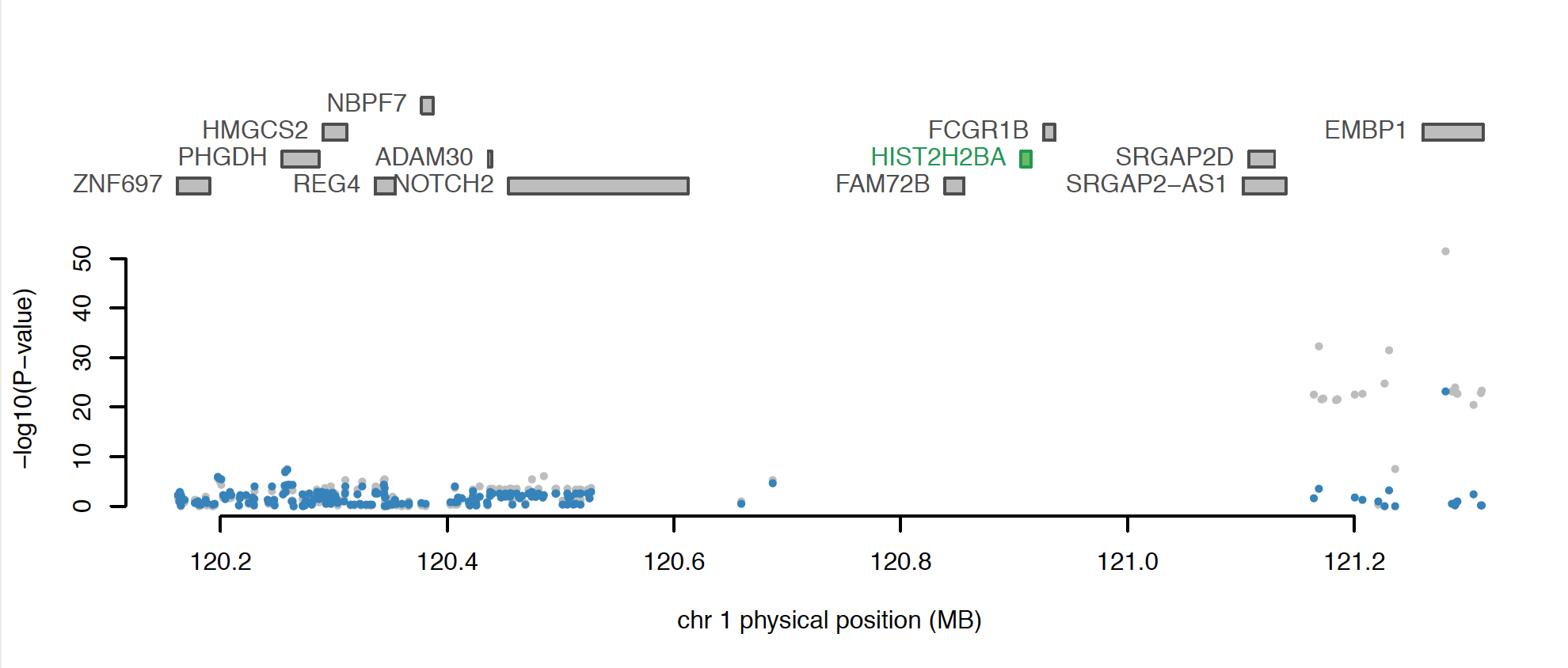


1. ZNF404


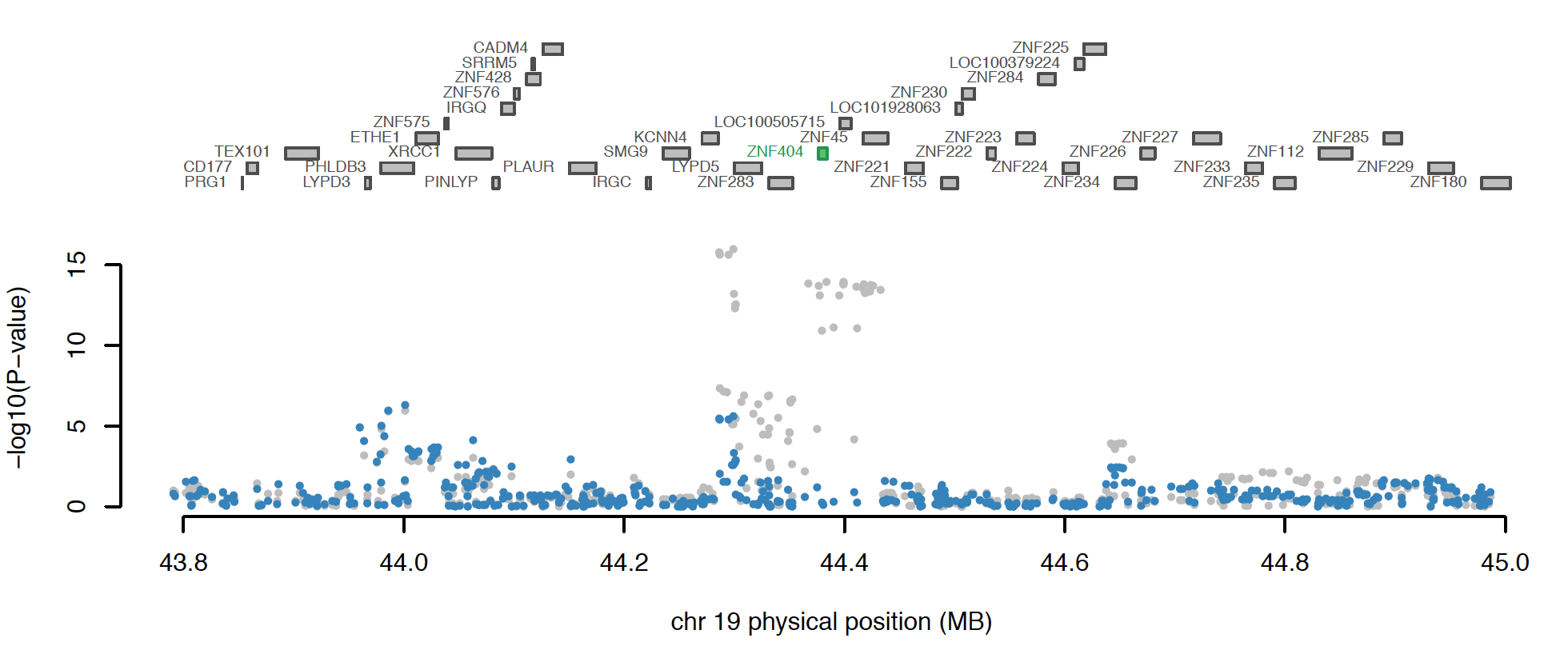


1. ZNF115


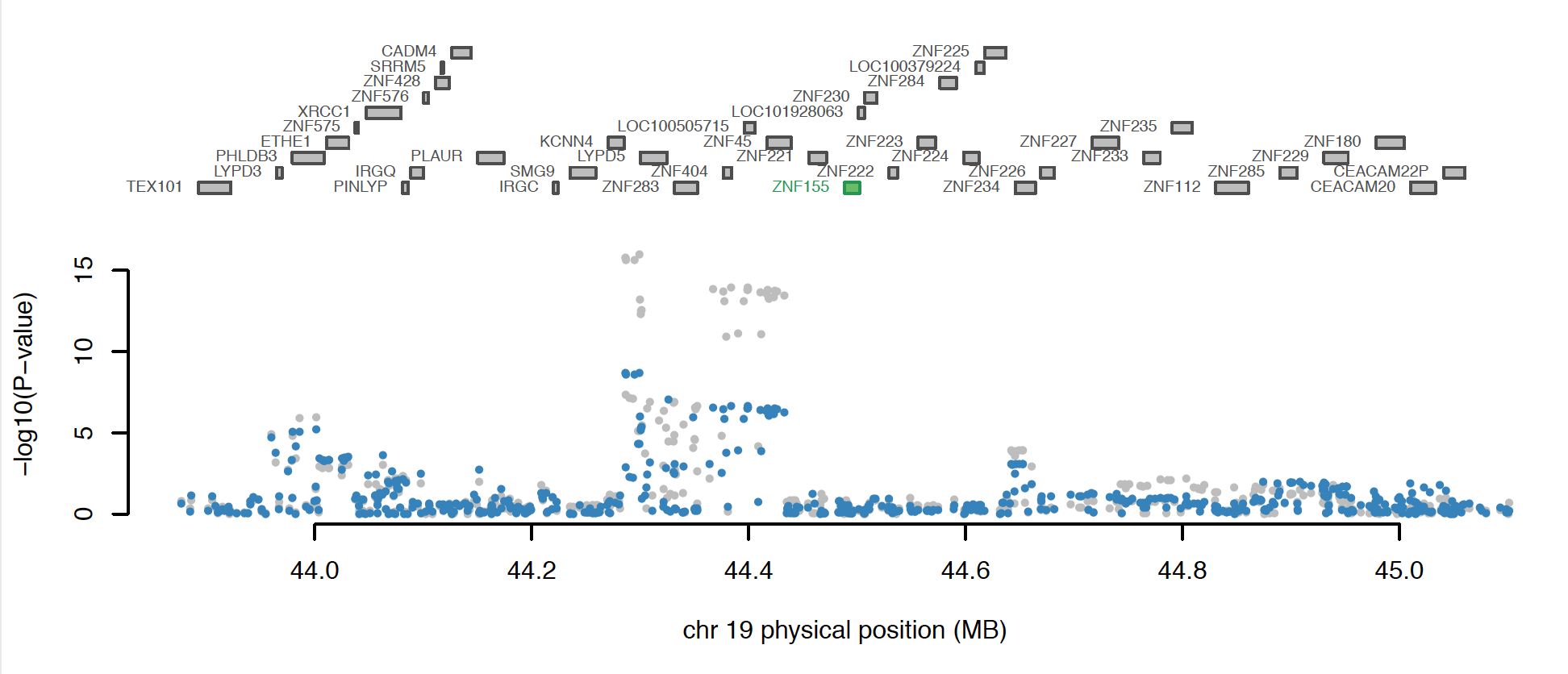


1. STXBP4
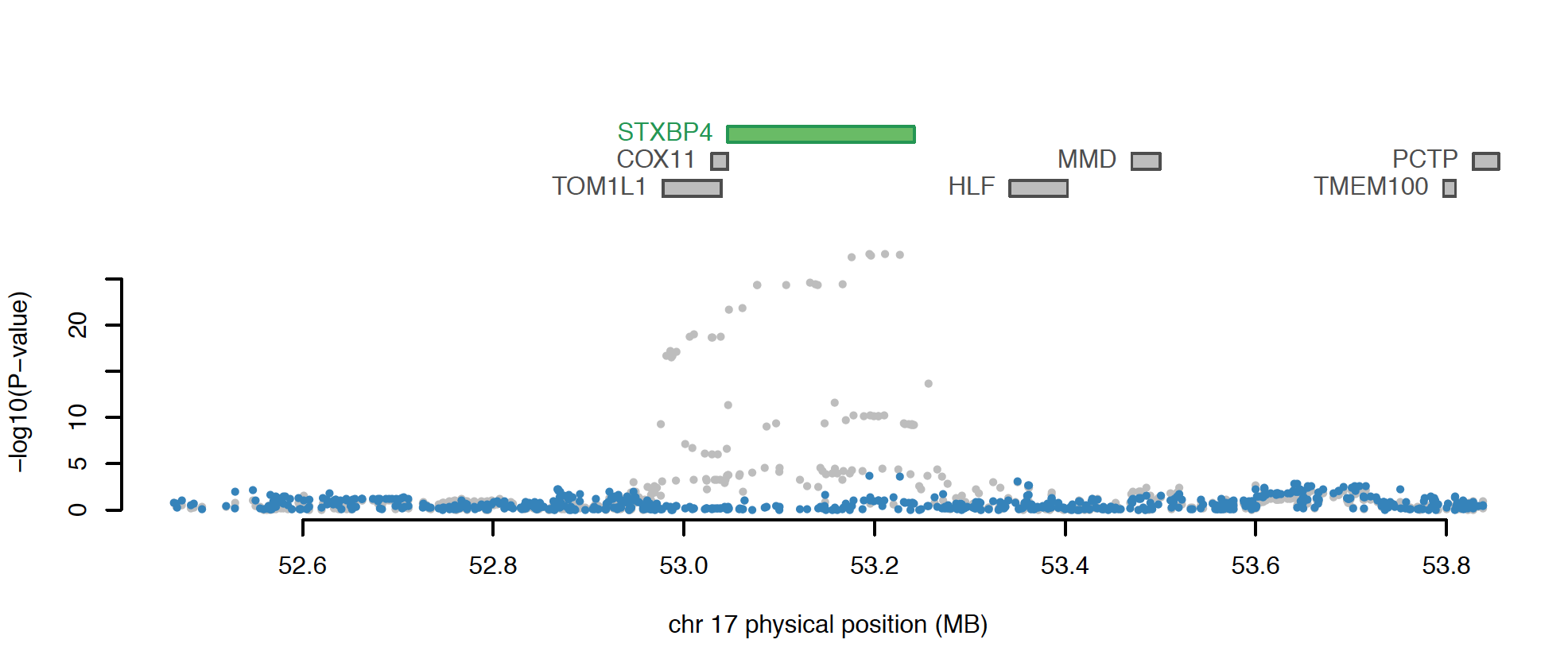

2. RP11-554A11.9


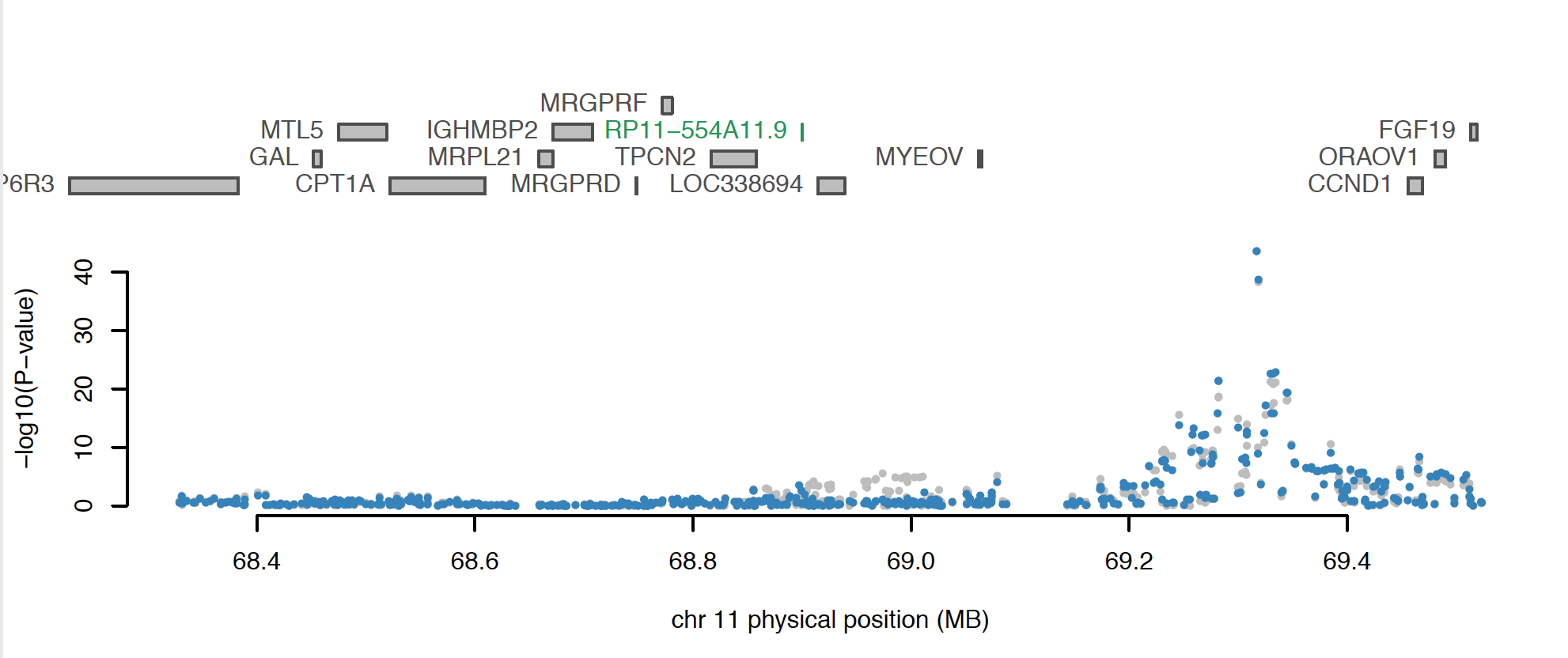


1. RP11-250B2.5


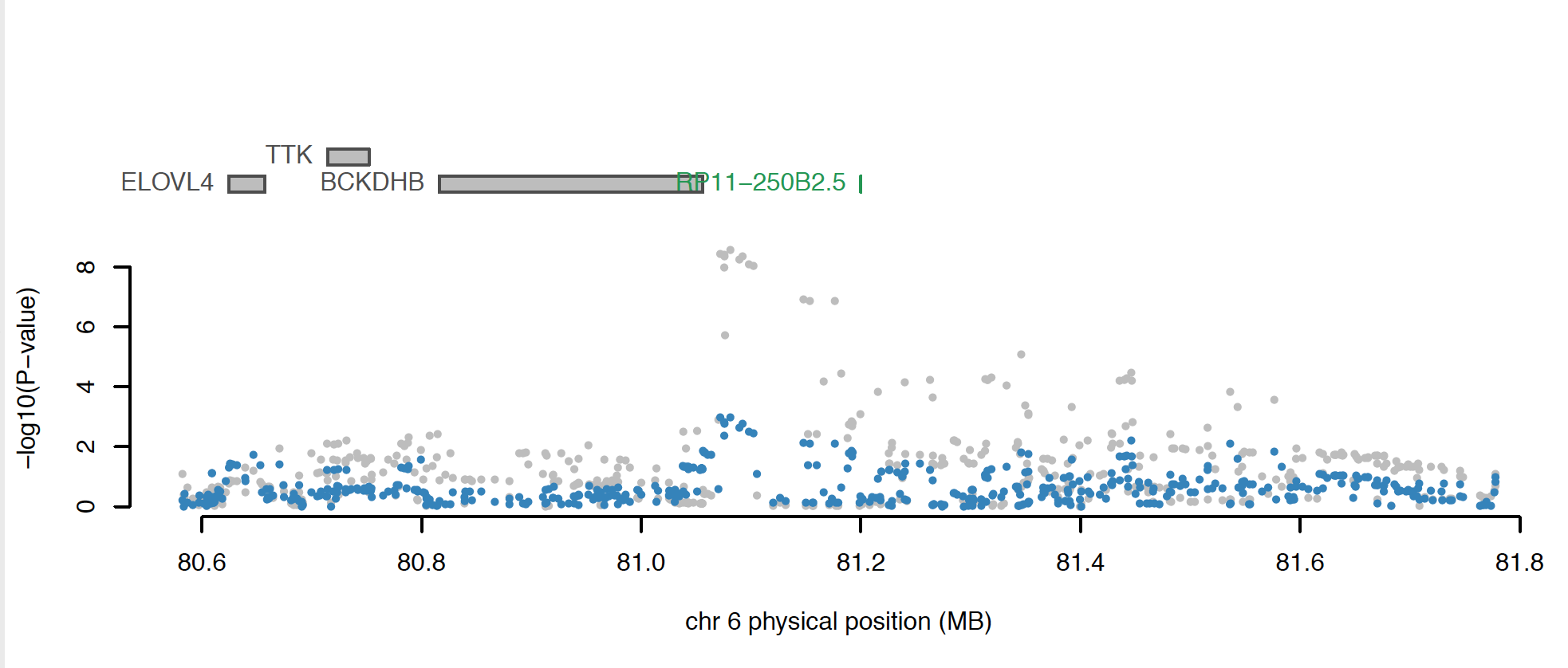


1. RP11-73O6.3


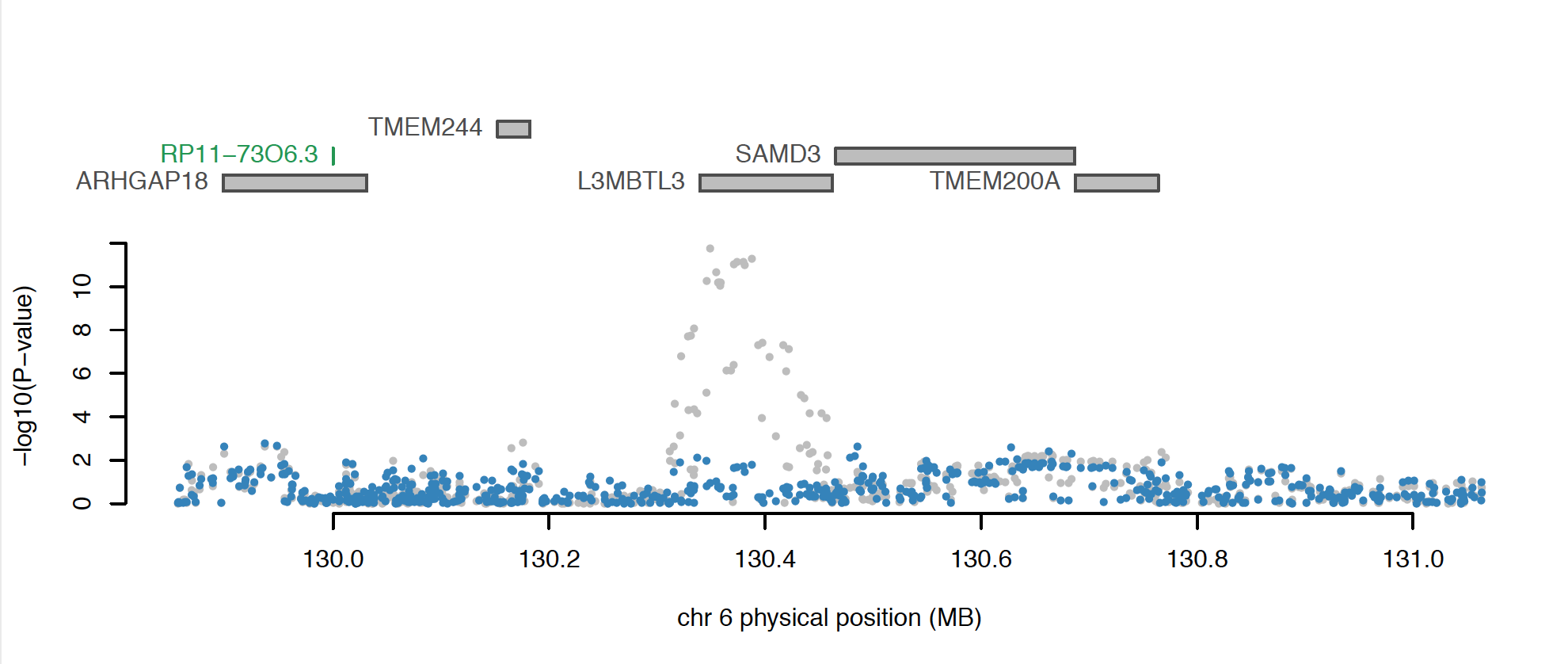


1. RP11-15A1.7


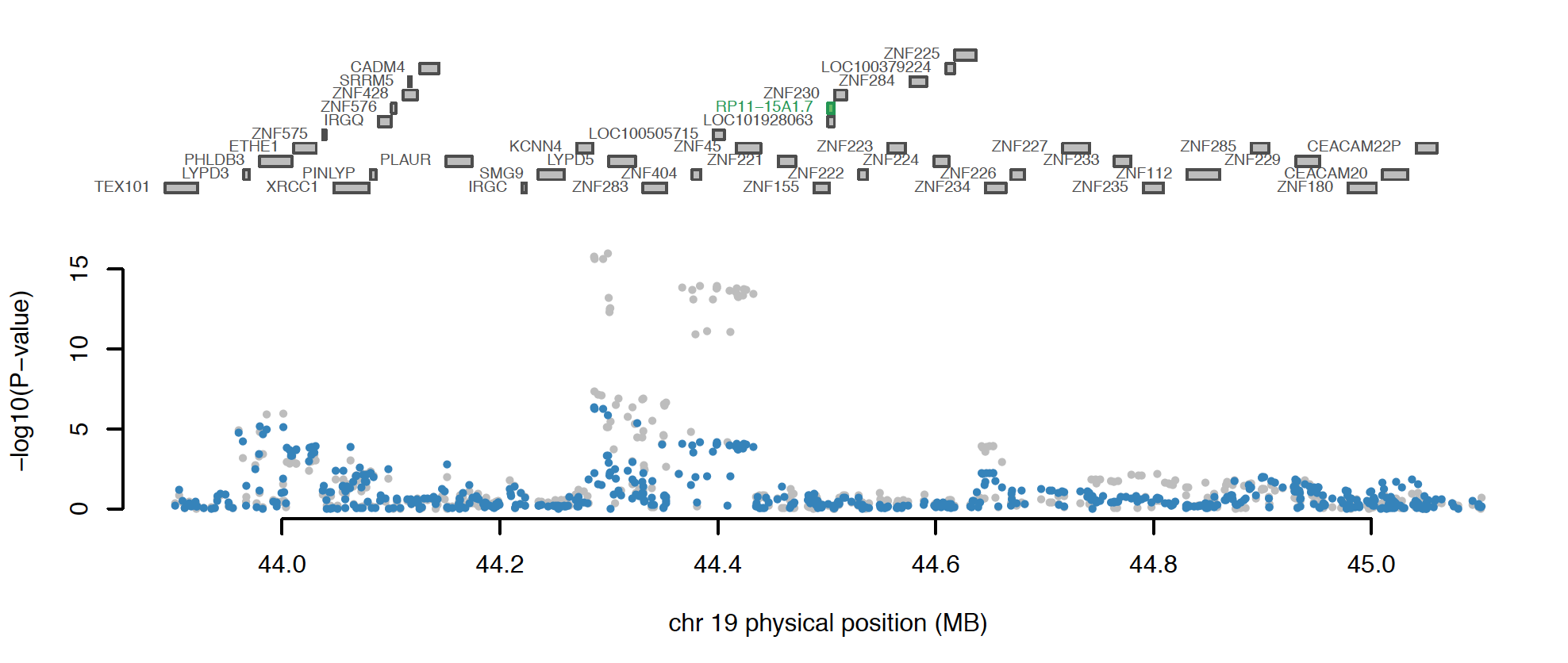


1. NUDT17


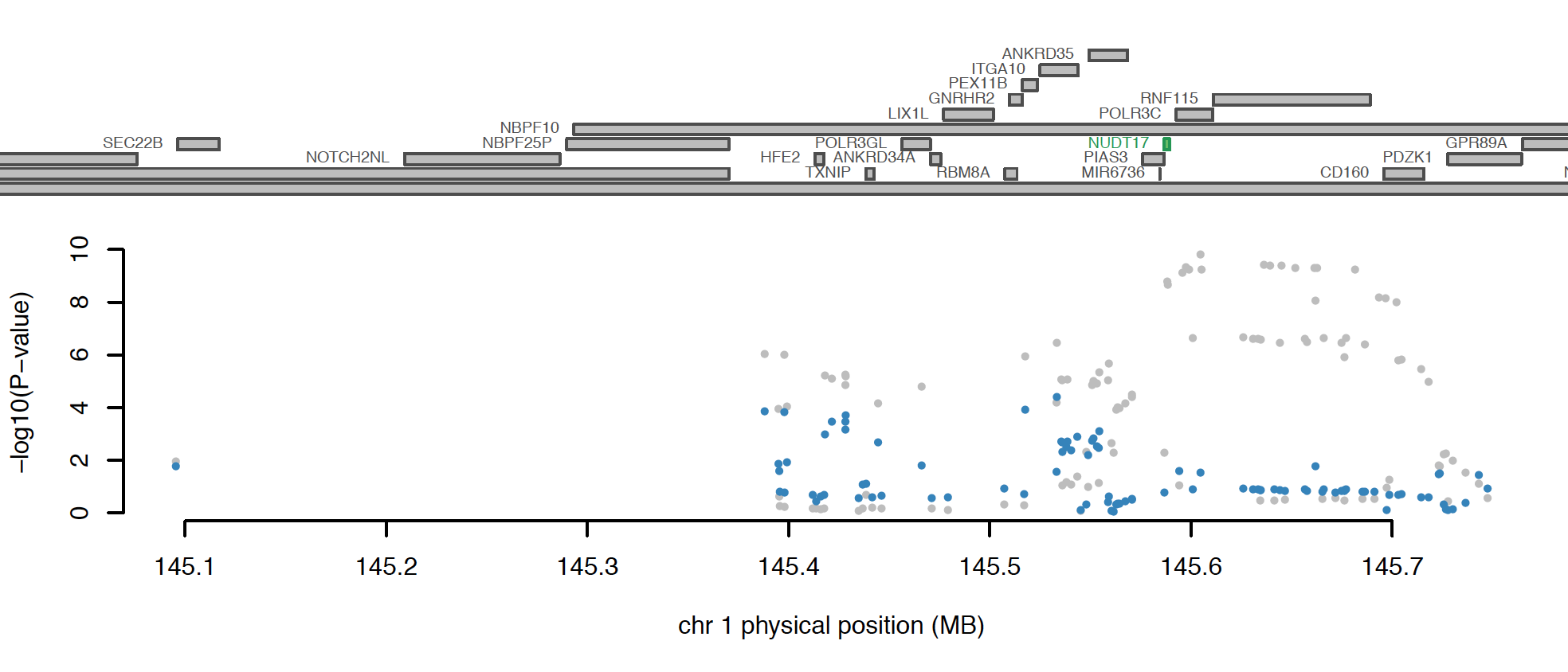


1. MRPL23-AS1


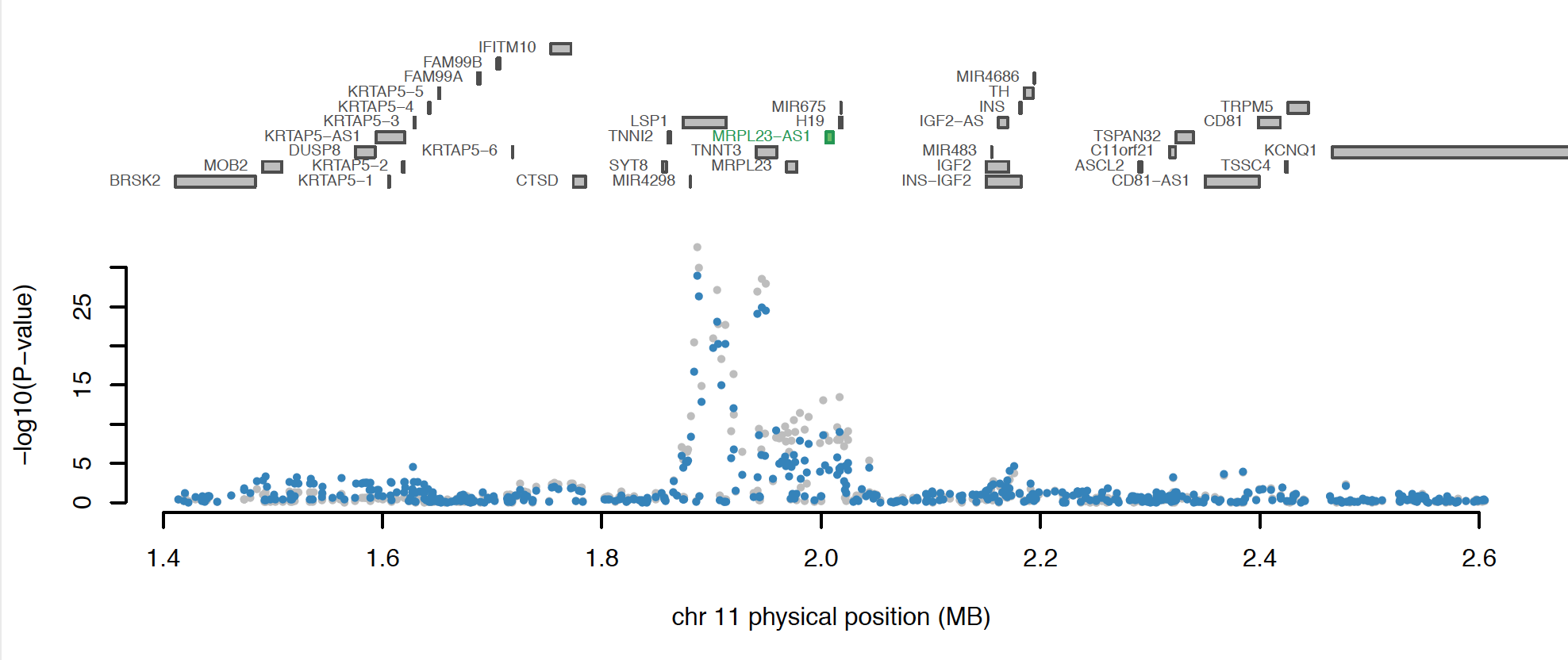


1. LRRC37A4P


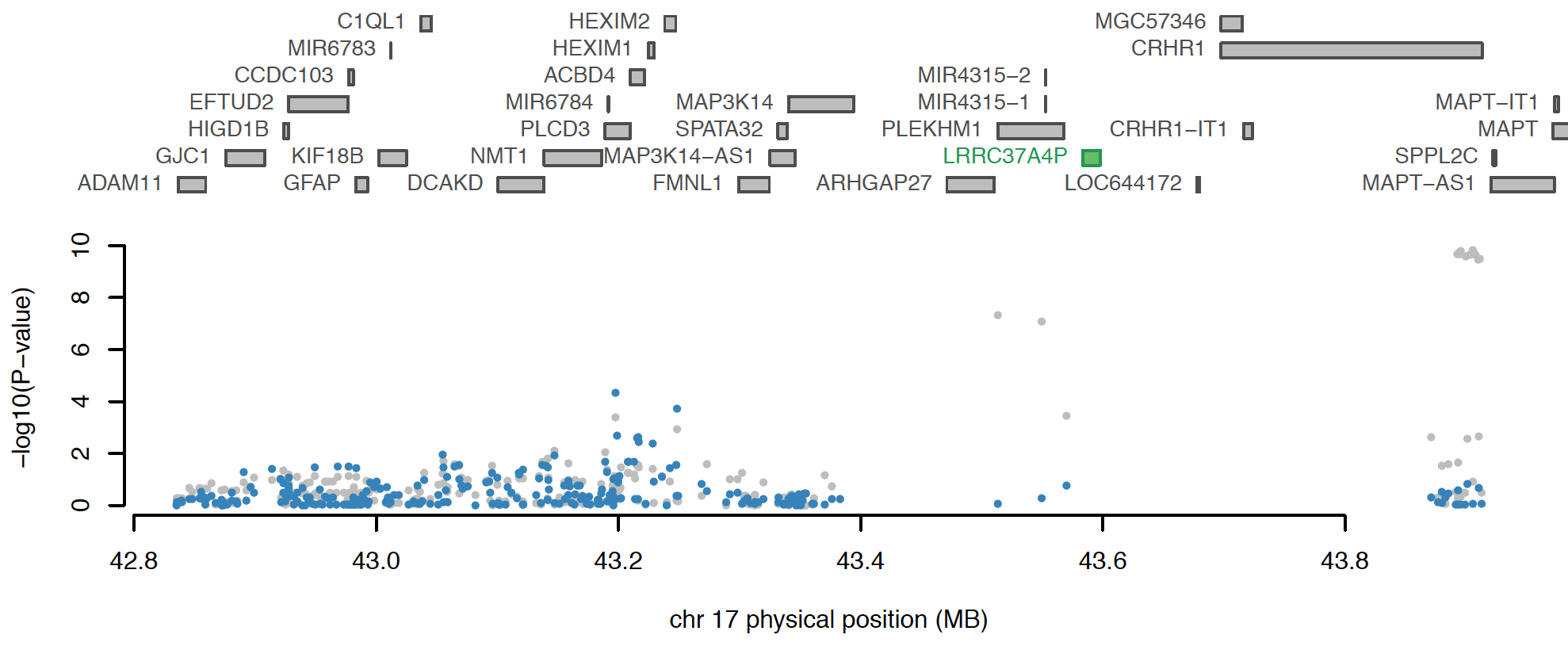


1. LRRC37A2


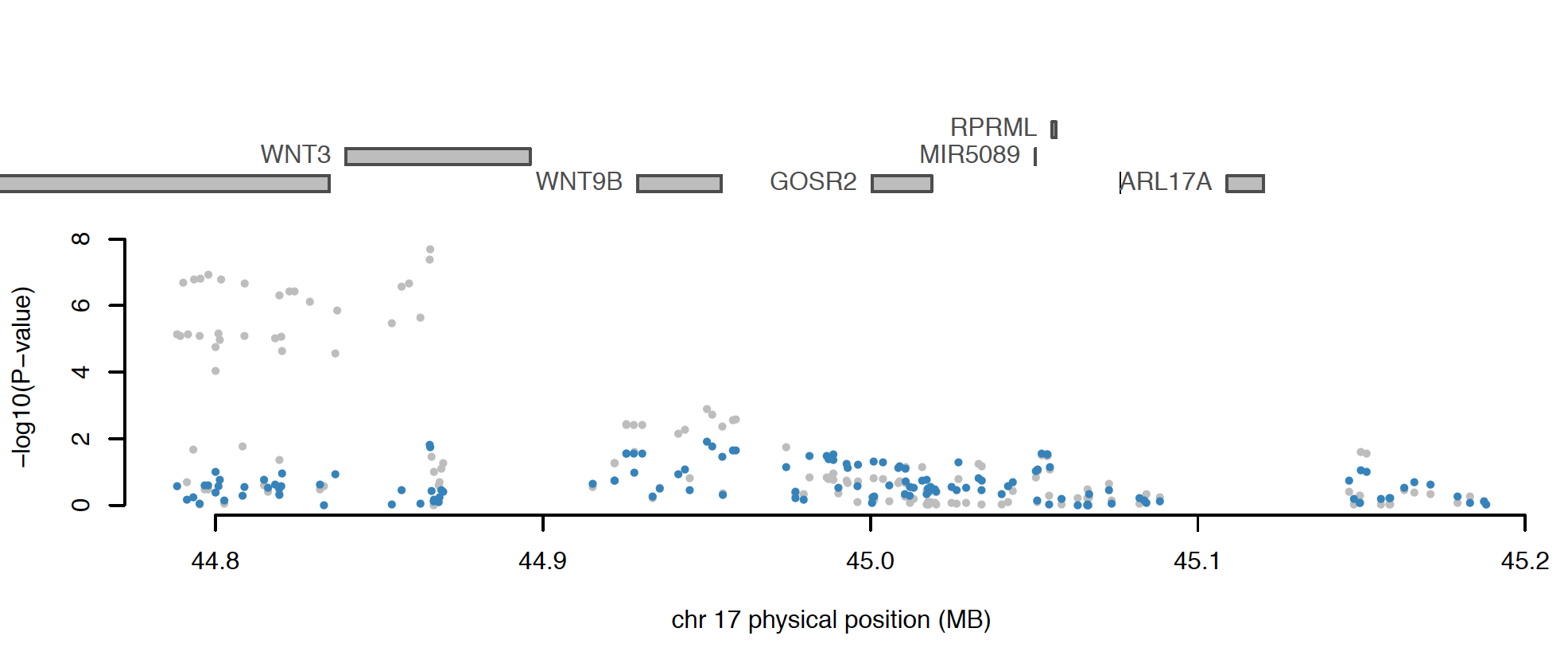


1. LRRC37A


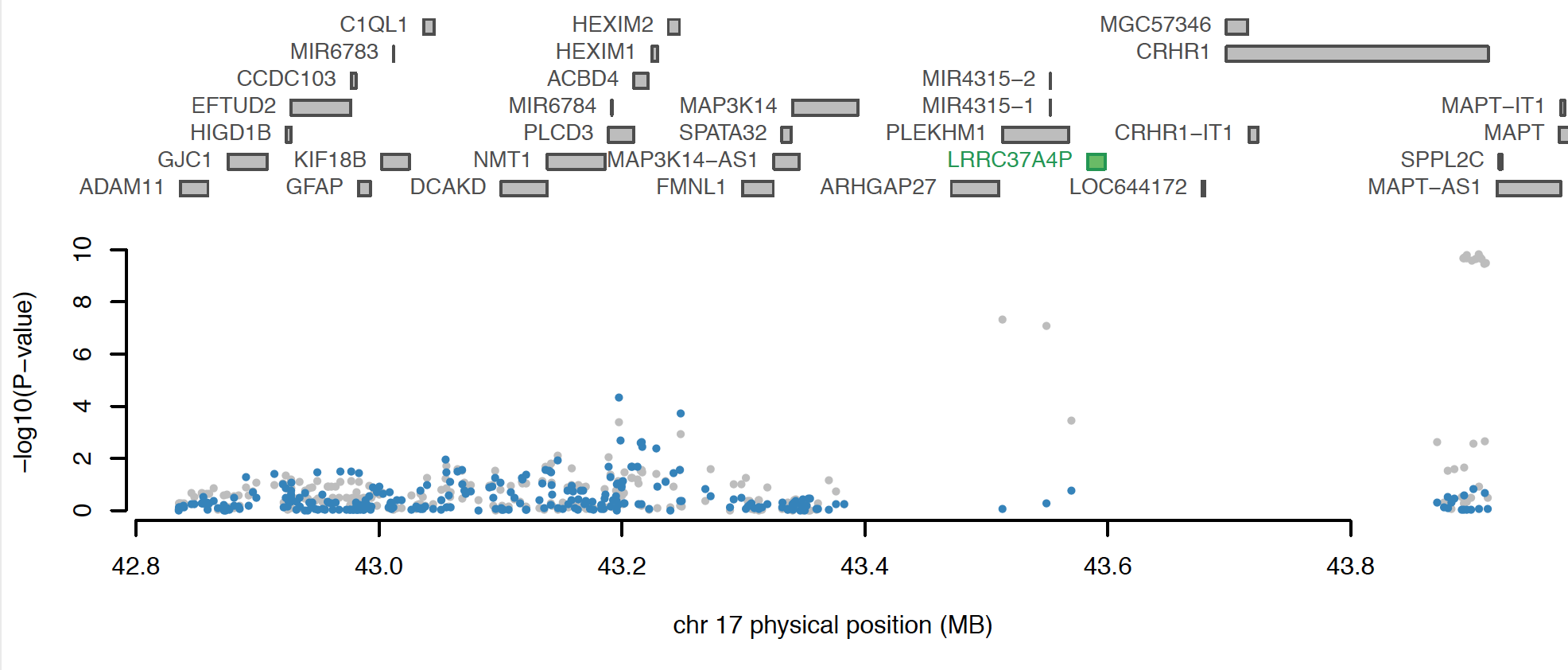


1. L3MBTL3


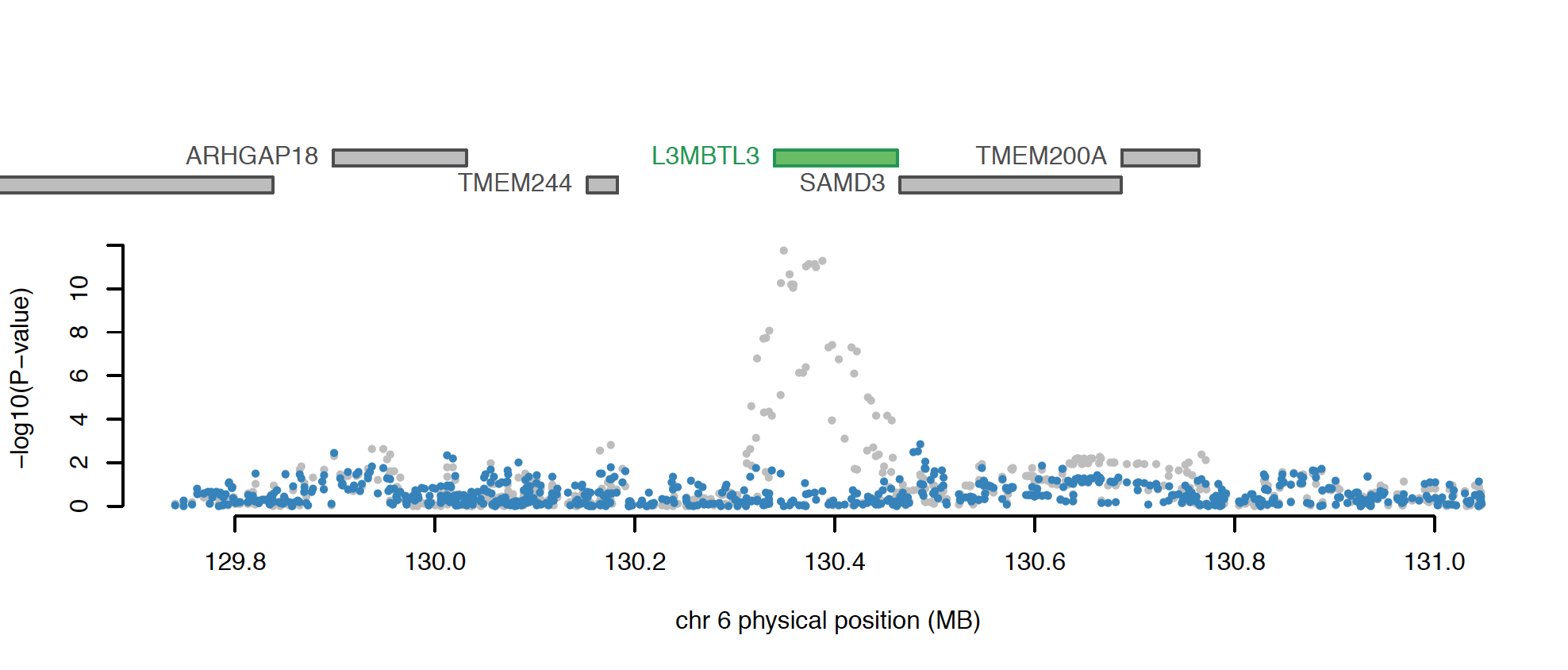


1. KANSL1-AS1


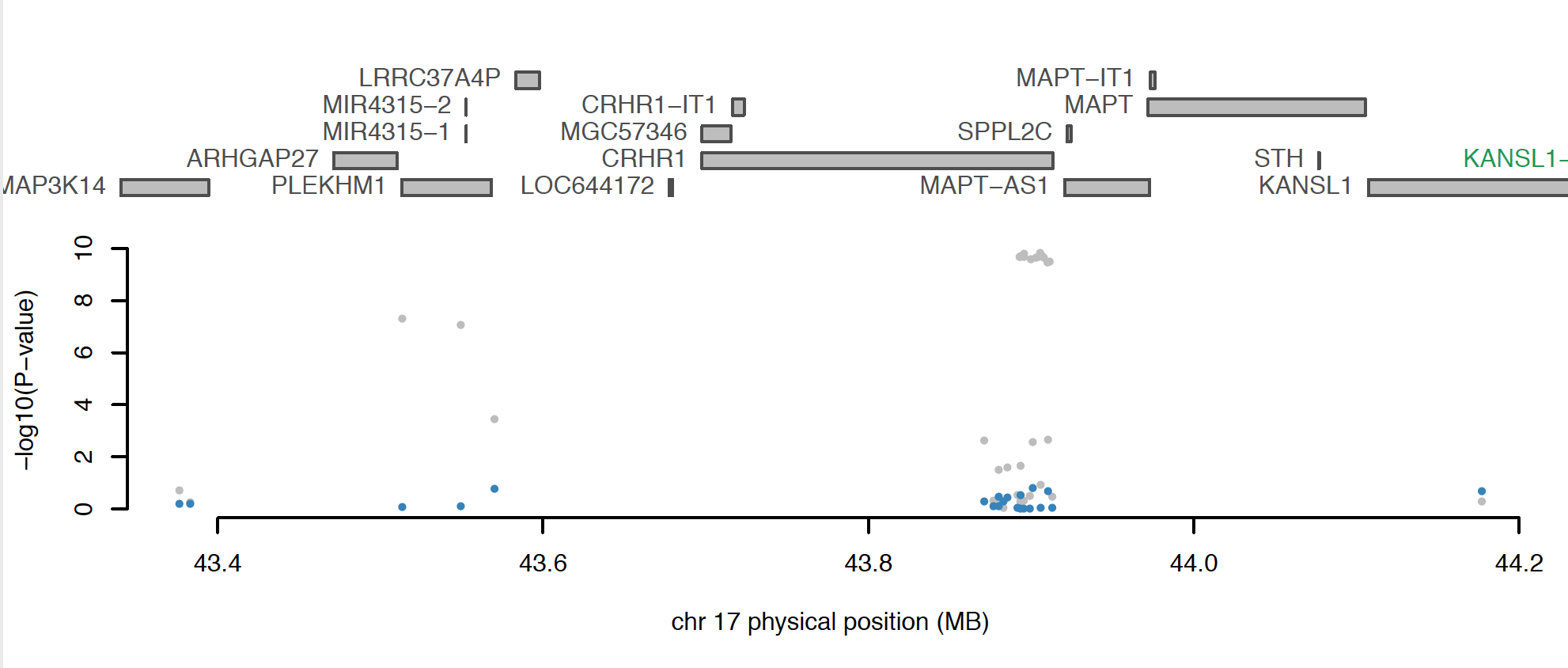


Supplementary Figure 2. Conditional analysis for overlapping region.

1. 2q33


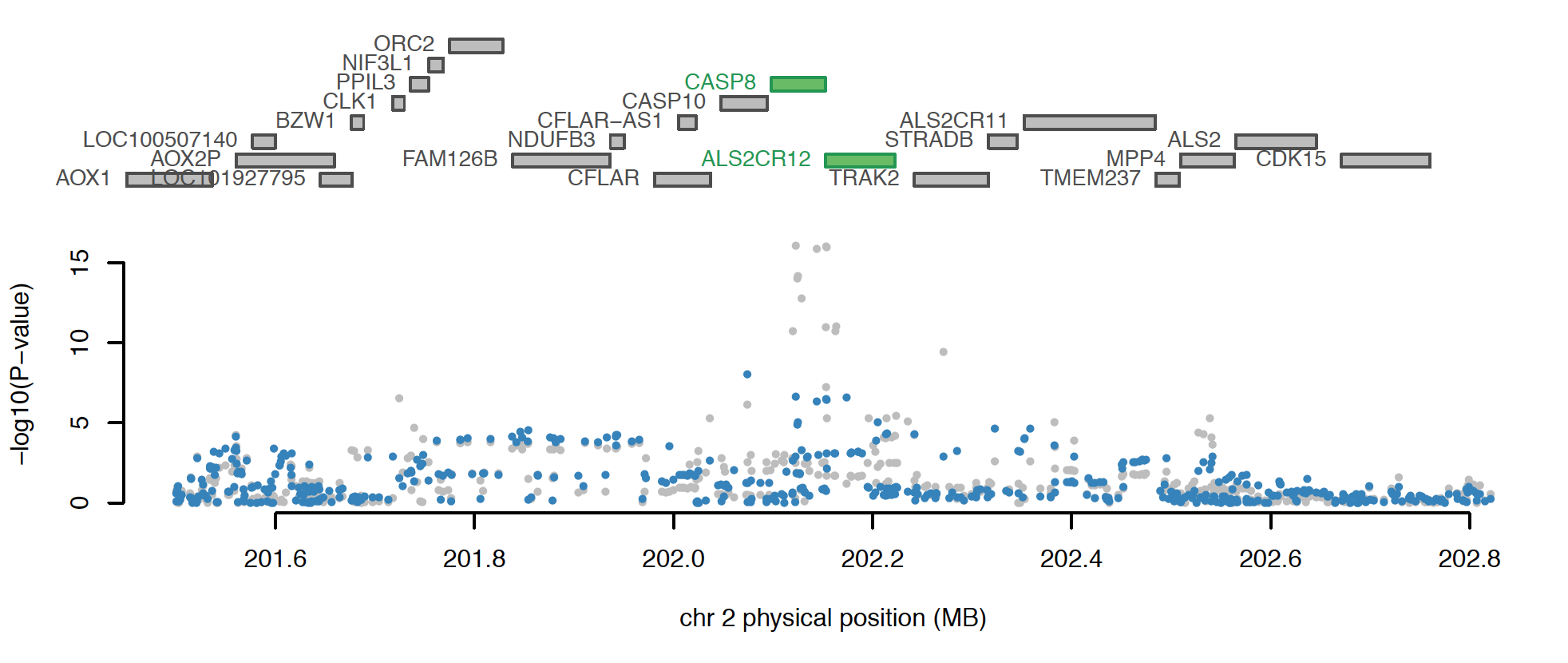


1. 5q14


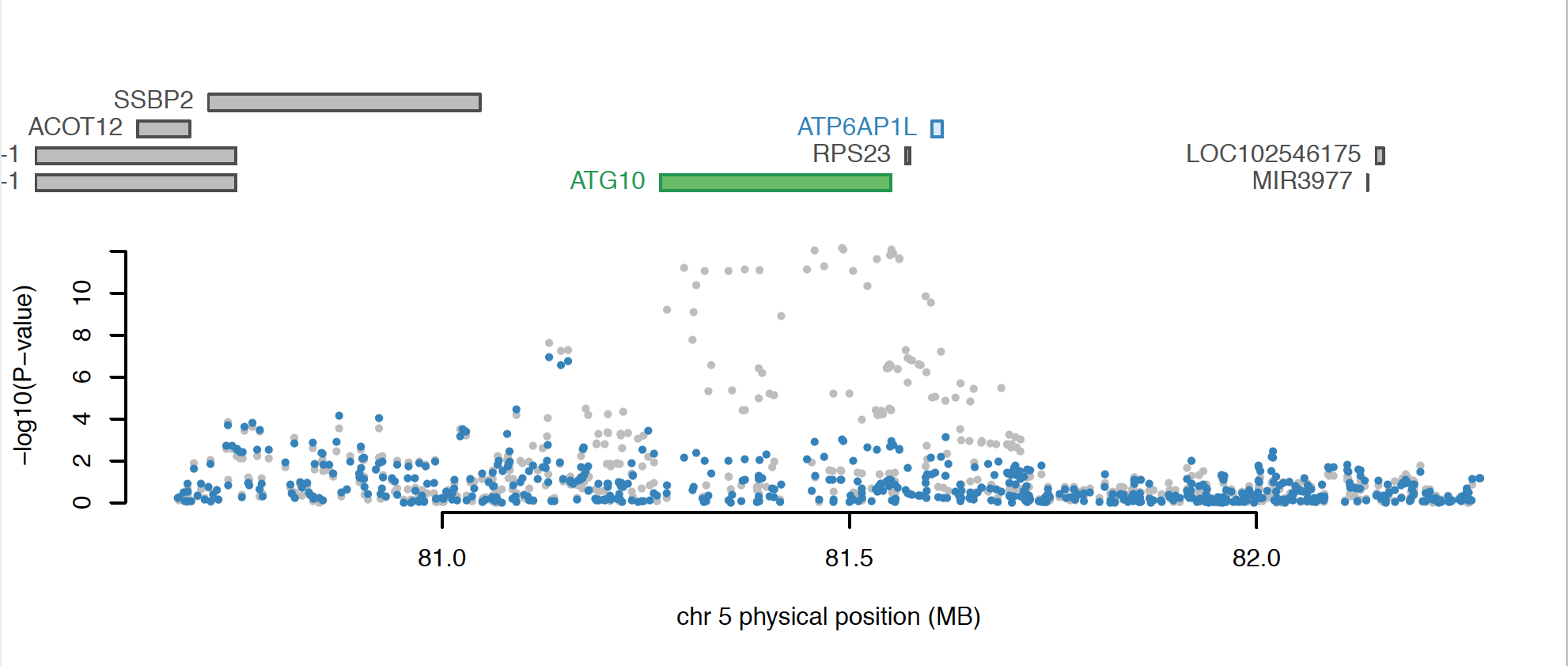


1. 6q22


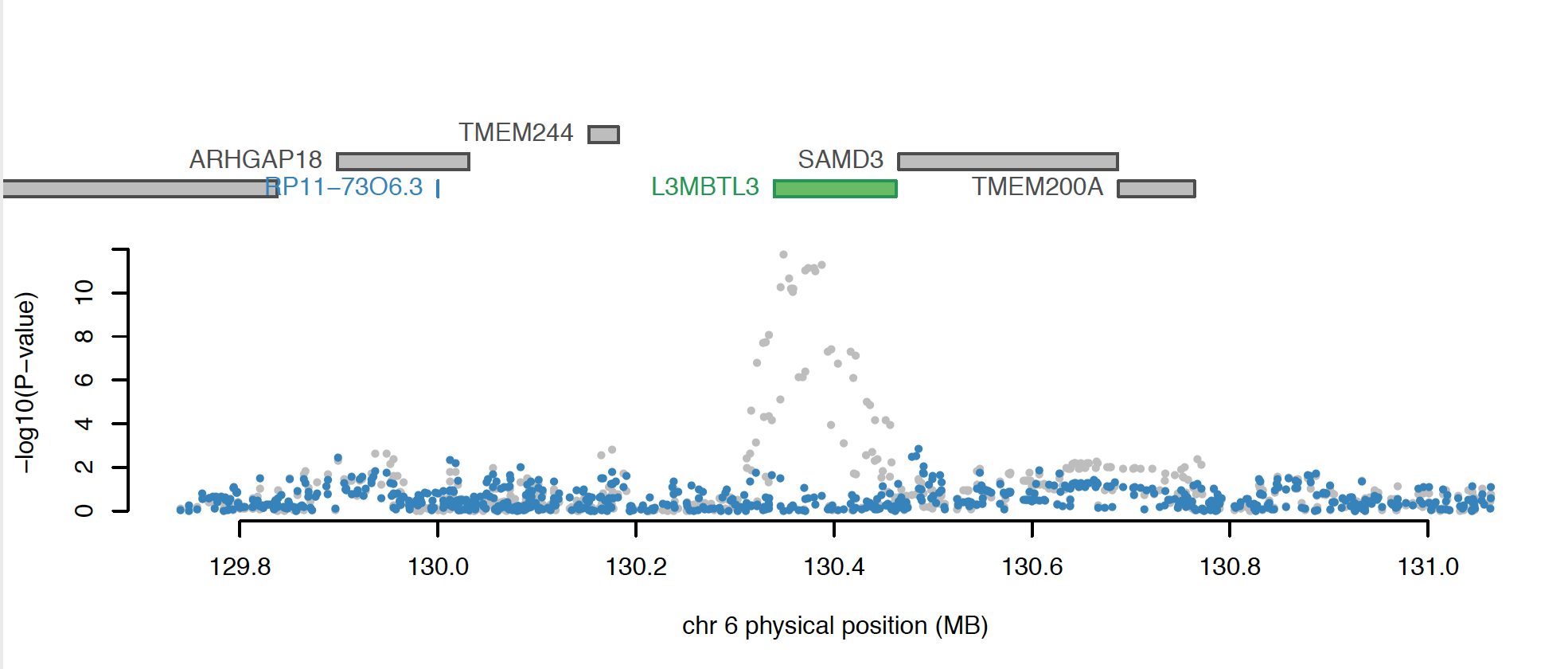


1. 15q24


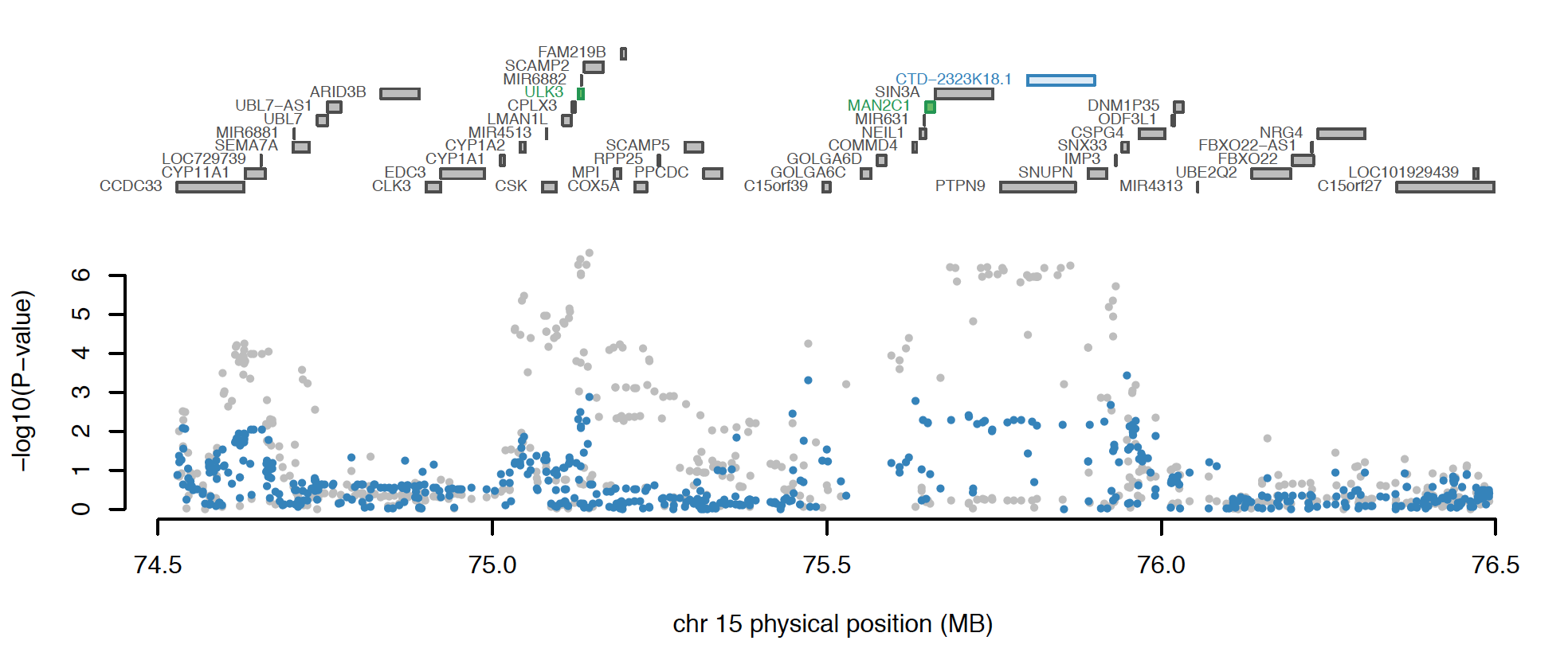


1. 17q21


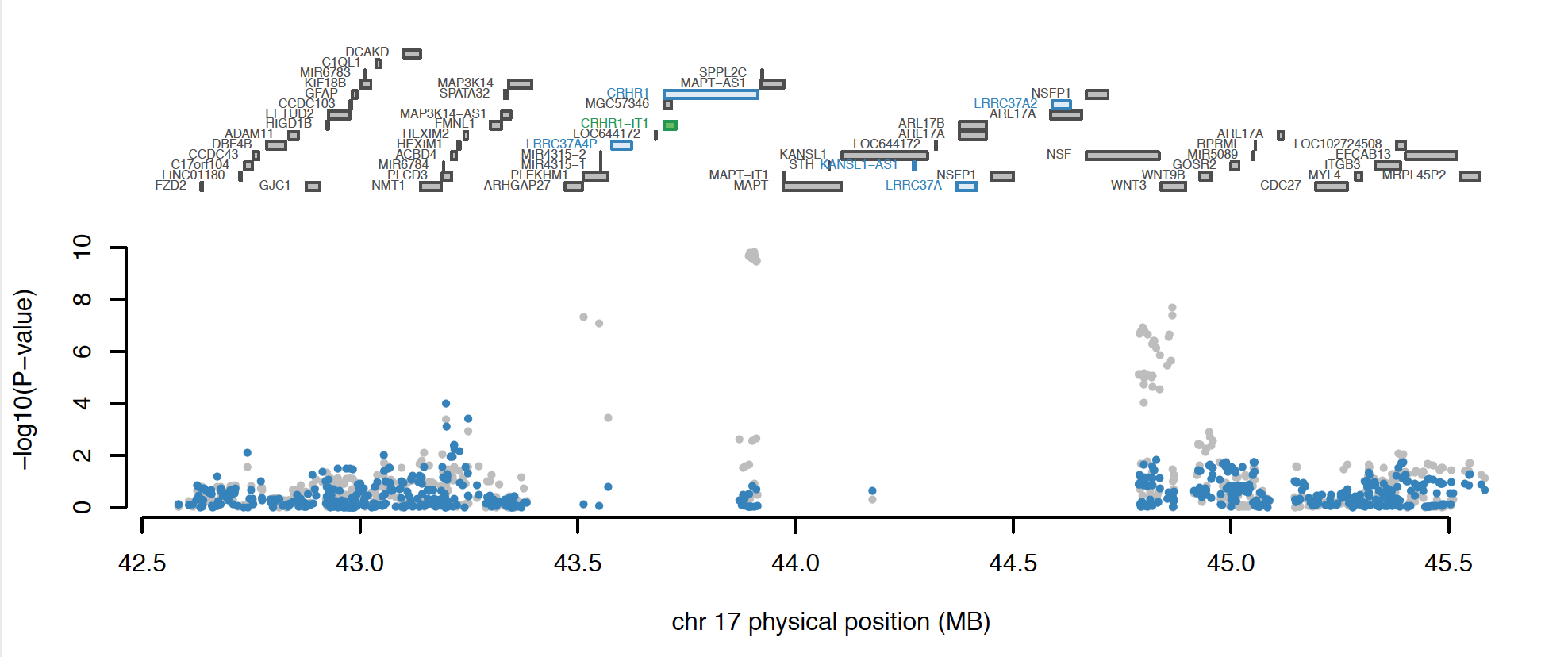


1. 19q13


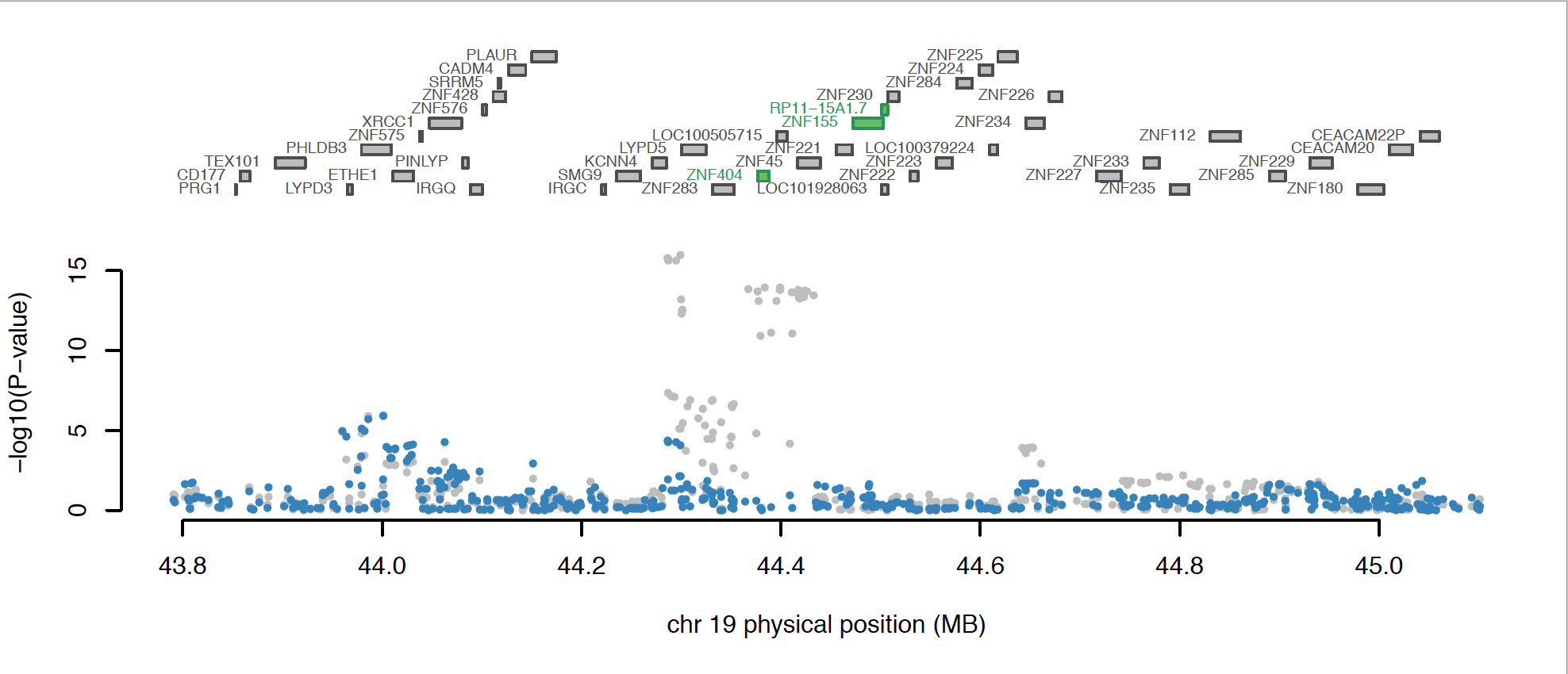

Supplement: supplementary material [file NIHMS1658893-supplement-supplementary_material.docx]
